# Neuroimmune Microenvironment Reprogramming via Immuno‐piezoelectric Transducers for Synergistic Stem Cell Therapy in Traumatic Brain Injury

**DOI:** 10.1002/adma.202512810

**Published:** 2025-10-01

**Authors:** Linlin Liang, Xin Li, Kai Hu, Pingqiang Cai, Jianwu Wang, Jing Yu, Shasha Wang, Yuwei Zhao, Changgeng Xu, Siwei Li, Hong Liu, Changyong Wang, Jin Zhou

**Affiliations:** ^1^ Beijing Institute of Basic Medical Sciences 27 Taiping Rd Beijing 100850 P. R. China; ^2^ State Key Laboratory of Crystal Materials Shandong University 27 Shandanan Road Jinan Shandong 250100 P. R. China; ^3^ Institute for Advanced Interdisciplinary Research University of Jinan Jinan Shandong 250022 PR China; ^4^ Digital Molecular Analytics and Science (IDMxS) Nanyang Technological University 59 Nanyang Drive Singapore 636921 Singapore; ^5^ The First School of Clinical Medicine Southern Medical University Guangzhou Guangdong 510515 P. R. China

**Keywords:** electrical stimulation, inflammatory, neural stem cells, piezoelectric nanomaterials, traumatic brain injury

## Abstract

Secondary traumatic brain injury (TBI) induces a pro‐inflammatory microenvironment that hampers neural stem cells (NSCs) therapy and tissue regeneration. To address this challenge, an immuno‐piezoelectric transducer have been developed to create an anti‐inflammatory immune microenvironment, deliver wireless electrical stimulation, and facilitate multimodal NSCs therapy. The immuno‐piezoelectric transducer drives the polarization of microglia towards the anti‐inflammatory M2 phenotype and secretion of anti‐inflammatory cytokines. This modulation significantly reduces the inflammatory response, creating an optimal microenvironment for NSCs survival. Furthermore, the wireless electrical stimulation generated by ultrasound facilitates NSCs differentiation into glutamatergic and GABAergic neurons, enhances neurite complexity, upregulates synaptic proteins expression and neural integration in injured regions. The multimodal therapy demonstrates superior outcomes in restoring structural integrity, improving functional, and enhancing behavioral action in TBI rat models. This study integrates piezoelectric with immunomodulation to reprogram the neuroimmune microenvironment, providing novel therapeutic paradigm for brain injury repair.

## Graphical abstract

A novel immuno‐piezoelectric transducer is innovatively developed. This device promotes neural stem cell (NSC) differentiation and maturation via ultrasound‐induced electrical stimulation and it amplifies the therapeutic efficacy of NSC in traumatic brain injury (TBI) by modulating the immune microenvironment. This piezoelectric transducer with “immune‐neuro” dual‐modulation functions, combined with NSC, provides a new strategy for nerve regeneration and repair.

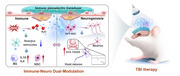

## 1 Introduction

Traumatic brain injury (TBI), a leading global cause of disability and mortality, presents significant clinical challenges due to neurodegeneration impairment and immune microenvironment imbalance caused by its pathological cascade.^[^ ^1^, ^2^, ^3^ ^]^ Although strategies such as stem cell transplantation, growth factor delivery, biomaterial scaffolds, and physical field intervention have shown potential in TBI repair, their therapeutic effects are still limited by the harsh pathological microenvironment and inherent defects in neural regenerative capacity.^[^ ^4^, ^5^, ^6^, ^7^ ^]^ Neural stem cells (NSCs) therapy, which differentiates and replaces damaged neurons to reconstruct new neural circuits, holds promise for the treatment of TBI.^[^ ^8^ ^]^ However, to date, therapeutic efficacy remains limited by three critical barriers: poor graft survival due to acute inflammatory storms and persistent immune microenvironment disorders, suboptimal integration and functional recovery of transplanted NSCs within complex pathological milieus, and unclear mechanisms underlying NSCs integration and repair under disease conditions.

Currently, to further enhance the therapeutic effect of stem cells, researchers are actively seeking synergistic strategies, such as the combined application of NSCs with biomaterials or physical field stimulation.^[^ ^9^ ^]^ Biomaterials have good effects on improving its differentiation efficiency and treatment.^[^ ^10^ ^]^ However, existing material systems generally lack the ability to actively regulate the pathological microenvironment and the ability to respond to external fields. Piezoelectric materials, as materials for external field interaction, can directly convert programmable ultrasound into local wireless electronic stimulation, providing unique opportunities for neural regulation.^[^ ^11^, ^12^, ^13^, ^14^ ^]^ This endogenous electrical stimulation has been confirmed to effectively promote the differentiation and maturation of NSCs and the growth of neurites, enhancing their in vivo repair ability.^[^ ^15^, ^16^, ^17^, ^18^, ^19^, ^20^ ^]^ Nevertheless, inorganic piezoelectric ceramics have poor biocompatibility and flexibility;^[^ ^21^, ^22^, ^23^, ^24^ ^]^ polymeric PVDF, while flexible, resists biodegradation;^[^ ^25^ ^]^ and degradable polymers (e.g., polylactic acid) generate acidic byproducts that induce cytotoxicity.^[^ ^26^ ^]^ Nanocellulose, a natural polymer material, offers advantages including abundant sourcing, low cost, biodegradability, biocompatibility, and intrinsic piezoelectricity.^[^ ^27^ ^]^ Yet, its inherently low piezoelectric coefficient falls short of neuromodulation requirements.^[^ ^28^ ^]^ Stretching can effectively produce self‐polarized cellulose piezoelectric nanofibers with a high β ratio, which significantly increases the piezoelectricity of piezoelectric materials.^[^ ^29^, ^30^ ^]^ Additionally, anisotropic fibers can simulate the oriented structure of regenerated natural nerves, supporting and guiding axon projection.^[^ ^10^, ^31^ ^]^

It is worth noting that piezoelectric materials are a “double‐edged sword” in the treatment of brain injury: on the one hand, their electrical stimulation promotes nerve regeneration; on the other hand, the reactive oxygen species (ROS) explosively generated in the acute phase after TBI not only directly damage nerves, recruit pro‐inflammatory microglia, exacerbate immune microenvironment imbalance, and seriously damage nerve regeneration and the survival of transplanted NSCs,^[^ ^32^, ^33^, ^34^ ^]^ but also generate additional ROS during the process of ultrasound of piezoelectric materials, further aggravating oxidative damage.^[^ ^35^ ^]^ Therefore, the anti‐inflammation and anti‐oxidation is a prerequisite for effective electrical stimulation and NSC‐based TBI therapies. It can not only solve the adverse factors in the use of piezoelectric materials but also improve the survival and therapeutic effect of stem cells through microenvironmental adjustment. Polydopamine (PDA) emerges as a highly promising biomaterial due to its enzyme‐mimetic properties, facile surface modification, high hydrophilicity, strong adhesion, excellent biocompatibility, and biodegradability.^[^ ^36^ ^]^ In recent years, studies have proved that PDA can realize the treatment of depression and ischemic stroke by scavenging free radicals and promoting microglia transformation.^[^ ^36^, ^37^ ^]^ Our team's previous studies revealed the positive effects of PDA on anti‐oxidation activity and macrophage polarization.^[^ ^38^ ^]^

**Figure 1:**
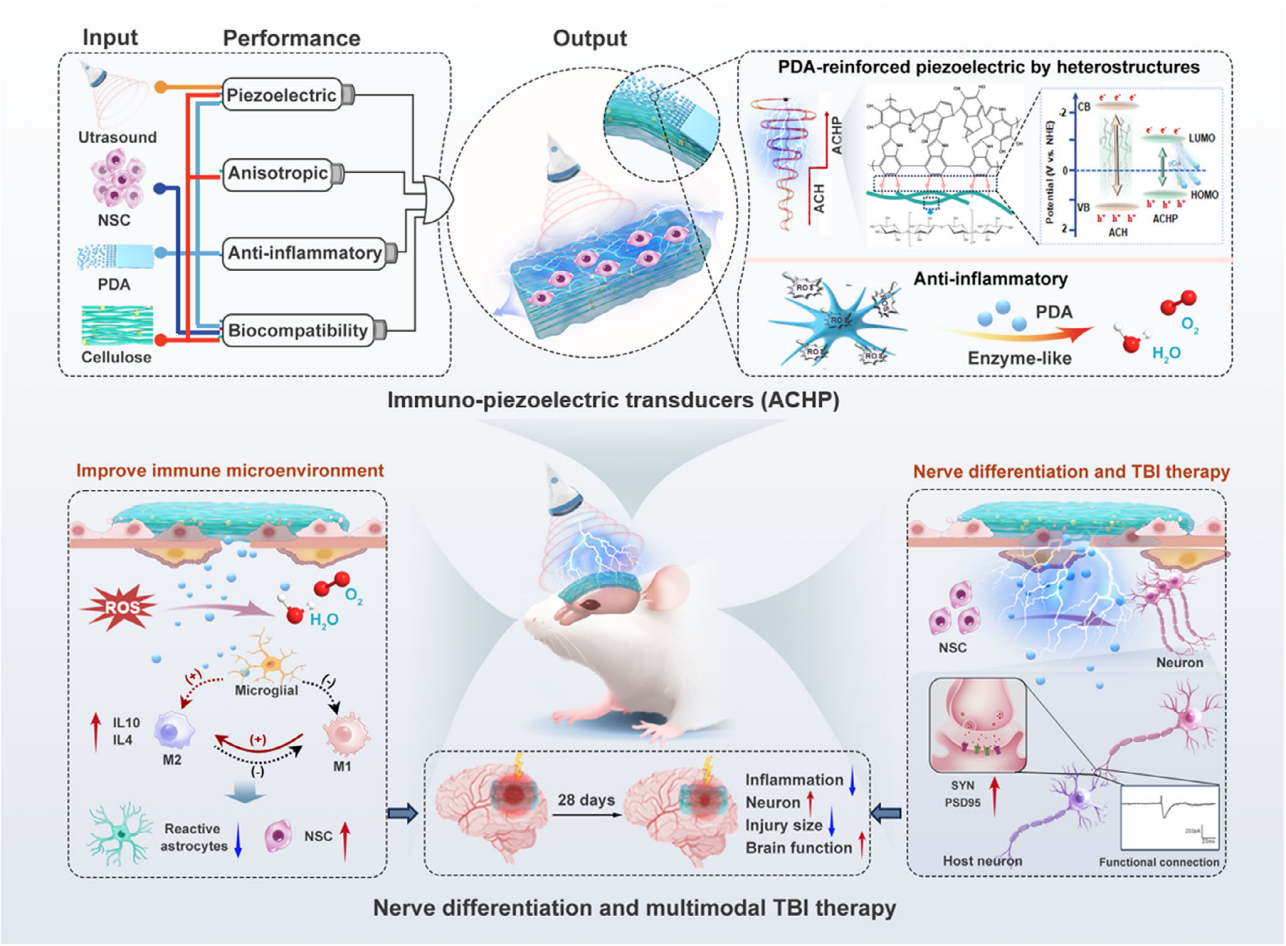
Schematic illustration of immuno‐piezoelectric transducers for nerve differentiation and regeneration. The immuno‐piezoelectric transducer is composed of anisotropic piezoelectric nanocellulose, anti‐inflammatory PDA coating and NSCs, which is activated by external programmable ultrasound to generate wireless electrical stimulation signals and promote the differentiation and development of NSCs. And its anti‐inflammatory properties improve the damaged immune microenvironment and improve the therapeutic effect of NSCs on TBI.

Addressing the critical challenge of spatiotemporal coupling between immune microenvironment dysregulation and electrophysiological modulation in current neural repair strategies, we innovatively developed an intelligent bioelectronic system: the immuno‐piezoelectric transducers (ACHP). This system integrates programmable ultrasound, stem cells, PDA coating, and anisotropic nanocellulose, demonstrating enhanced piezoelectric properties, stable electrical output, anti‐inflammatory efficacy, and superior cell adhesion. Ultrasound‐triggered piezoelectric signals promote glutamatergic/GABAergic neuronal differentiation via NSCs regulation, while the sustained polydopamine coating scavenges ROS byproducts generated during piezo stimulation and facilitates M2 microglial polarization alongside anti‐inflammatory factor secretion, and further improves their piezoelectric performance. This “immuno‐electrical” bidirectional synergistic mechanism ultimately drives neural circuit reconstruction and functional recovery in injured regions (**Figure** **1**), which establishing a new paradigm for intelligent bioelectronic systems in neural regeneration.

## 2 Results and Discussion

### 2.1 Construction and Characterization of Immuno‐Piezoelectric Transducers

**Figure 2:**
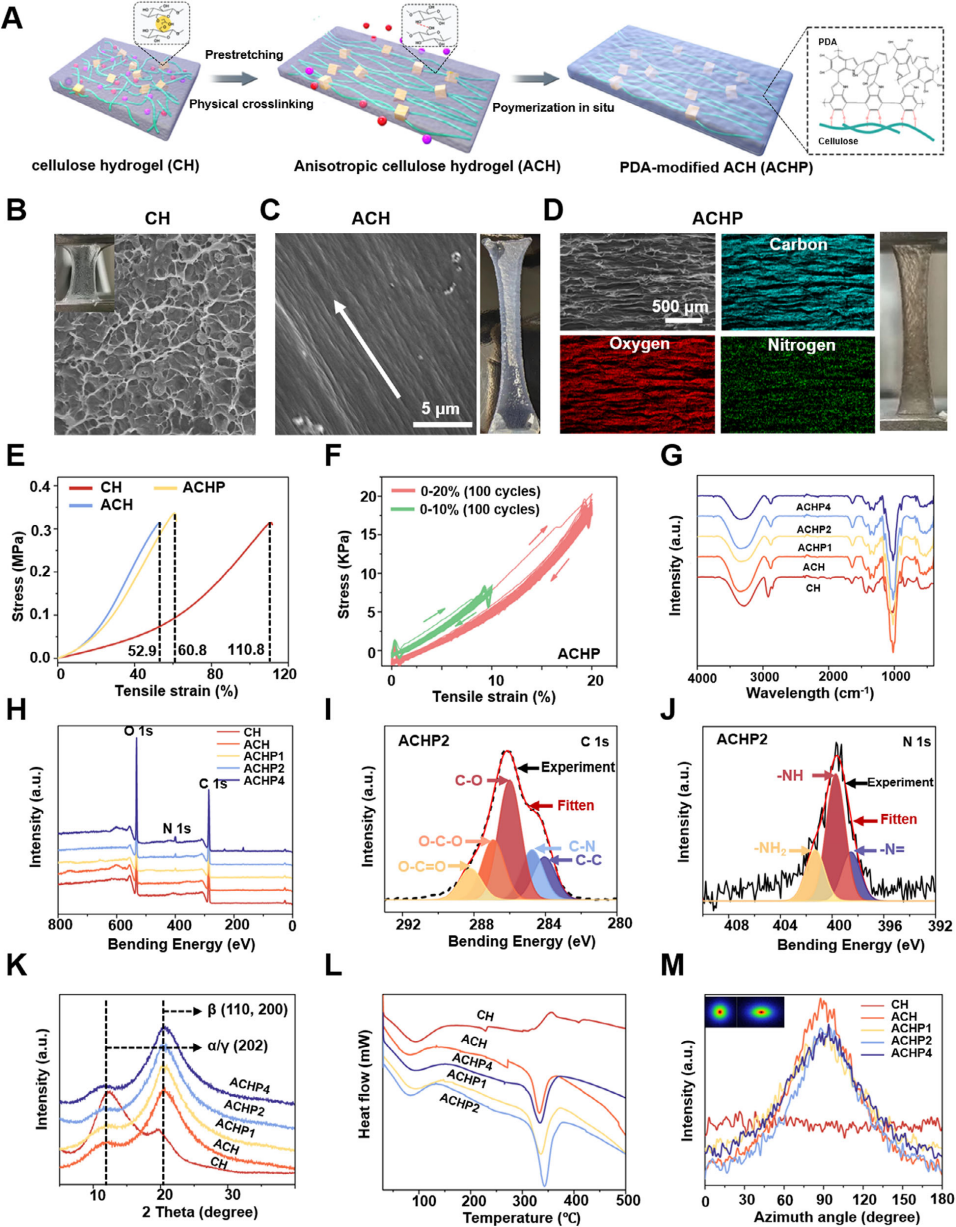
Characterization of immuno‐piezoelectric transducers. A) Fabrication of anisotropic anti‐inflammatory piezoelectric hydrogels. B) SEM images of CH (the actual picture of CH in the upper left corner), and C) SEM images of ACH, the white arrow is the ordered direction of the fiber surface. D) EDS mapping of C (blue), O (red), and N (green) elements distribution in ACHP. E) Stress–strain curve of piezoelectric hydrogel. F) fatigue resistance of ACHP under loading–unloading cycles. G) FTIR spectroscopy, L) DSC thermogram, and K) XRD of piezoelectric hydrogels with different PDA contents. H) Full XPS scanning spectra of different piezoelectric hydrogels. XPS I) C1s, and J) N1s spectra of ACHP. M) An azimuth‐integral intensity distribution curve of the SAXS mode, where 0° represents vertical direction, inset is the 2D SAXS pattens of CH and ACH, ACH formed a sharp, elongated equatorial streak, and the ring strength was anisotropic.

Anisotropic piezoelectric hydrogels composed of nanometer‐sized arrayed nanofibers were prepared by double crosslinking and prestretching methods (**Figure** **2**A). Initially, the filter paper scraps were dissolved in an alkali/urea solution at a low temperature to form a transparent cellulose solution (Figure S1A, Supporting Information). In this system, the cellulose chain and alkali‐urea‐water cluster were combined at a low temperature to form an inclusion complex through hydrogen bond formation, resulting in cellulose dissolution.^[^ ^39^ ^]^ Atomic force microscopy (AFM) confirmed the cellulose's nanofiber structure (Figure S1A, Supporting Information).

Nanocellulose contains many hydroxyl groups, and a small amount of epichlorohydrin (ECH) can cause the hydroxyl groups in cellulose to react in alkali/urea aqueous solution to synthesize chemically cross‐linked cellulose hydrogels (LCCs). This crosslinking reaction proceeds via epoxy ring‐opening and subsequent etherification of the cellulose hydroxyl groups under alkaline conditions, as illustrated schematically in Figure S1D (Supporting Information) and further detailed in the supporting information. To achieve anisotropic nanocellulose hydrogels with high tensile strength and low modulus, we tailored the fracture strain of LCCs by adjusting the ECH to anhydro glucose unit (AGU) molar ratio in the crosslinking reaction, resulting in varying mechanical properties across LCC‐1 to LCC‐7 (Figure S1E, Supporting Information). LCC‐3, with an ECH/AGU ratio of 1.5, exhibited the highest fracture strain (215%) and an extremely low modulus (4.2 kPa) (Figure S1F, Supporting Information), and demonstrated excellent shape‐recovery performance (Figure S1C, Supporting Information). LCC‐3 was thus chosen for fabricating anisotropic cellulose hydrogels (ACH). LCC‐3, prestretched to induce directional structure, rapidly resumed their original shape upon removal of external forces (Figure S1G, Supporting Information). After prestretching ACH to a specific degree (Figure S2, Supporting Information) by leveraging cellulose's strong self‐aggregation tendency, it was immersed in sulfuric acid to dissolve the base/urea solvent shell around cellulose chains, permanently fixing the rearranged structure through physical cross‐linking via hydrogen bonding, thus yielding distinct ACH.

According to the SEM characterization shown in Figure 2B, CH without prestretching was morphologically uniform and had a porous structure. The ACH gradually formed oriented structures along the prestretching direction with an increase in the prestretching ratio (Figure S3A, Supporting Information). ACH had a remarkable orientation (Figure 2C). The element distribution map of ACH showed that O and C were evenly distributed throughout the material (Figure S3B, Supporting Information). Therefore, ACH was used as the substrate material for the subsequent material modification. To improve the piezoelectric properties and biocompatibility of ACH, a layer of polydopamine (PDA) was in situ polymerized on ACH and called ACHP (Figure 2D). The composition of the ACHP hydrogel was determined by the distribution of elements. The results showed that O, C, and N were evenly distributed throughout the matrix of the ACHP hydrogel, which also indicated that the PDA modification was successful.

Tensile tests were performed on various hydrogels to evaluate the effects of stretching and surface modification on their mechanical properties. As shown in Figure 2E, the mechanical strength of ACH increased with prolonged prestretching time, accompanied by a rise in both tensile strength and elastic modulus, while the fracture strain decreased markedly to 49.6%. After modification with PDA, the tensile strength remained largely unchanged, whereas the fracture strain increased significantly. These results indicate that the PDA coating reinforces the cellulose matrix through robust interfacial bonding (e.g., covalent and hydrogen bonds), facilitating efficient stress transfer without compromising the intrinsic strength of the pre‐aligned network. Simultaneously, reversible hydrogen bonds at the PDA‐cellulose interface act as sacrificial bonds that dissipate energy under deformation, thereby enhancing the ductility and toughness of the hydrogel‐a mechanism consistent with the sacrificial bond theory widely observed in tough hydrogels.

Unless otherwise specified, the ACHP mentioned below refers to the hydrogels (ACHP2) modified with a dopamine concentration of 2 mg mL^−1^. The cyclic stress–strain curve of ACHP hydrogel showed that the ACHP hydrogel had prominent hysteresis, and the stress increased with increasing strain (Figure 2F). Fatigue resistance is an important property of hydrogels. The results of fatigue resistance tests of CH, ACH, and ACHP after 100 loading and unloading cycles showed that the hydrogel had no prominent plastic deformation or strength degradation after the first stretching cycle in the range of 10% to 20%. Quantitative analysis further demonstrated that ACHP maintained a higher percentage of residual strength and less stiffness degradation after 100 loading–unloading cycles, indicating significantly superior fatigue resistance over both CH and ACH (Figures S4A,B, Supporting Information). These findings indicated that CH, ACH, and ACHP had good antifatigue properties (Figure S1H, Supporting Information).

To elucidate the influence of PDA on anisotropic hydrogel formation, ACHP were synthesized at varying dopamine concentrations (1, 2, and 4 mg mL^−1^). SEM analysis (Figure S3C, Supporting Information) revealed that at the lowest PDA concentration, ACHP1 displayed disconnected nanoparticles due to insufficient PDA for continuous film formation. At 2 mg mL^−1^, a continuous, smooth film encapsulated ACH, yet higher concentrations led to PDA nanoparticle aggregation on ACH's surface. To further verify the uniformity and thickness of the PDA coating, TEM characterization of the ACHP cross‐section revealed a PDA layer with a consistent thickness of ≈55 nm (Figure S5, Supporting Information). FTIR spectroscopy confirmed cross‐linking and hydrogen bonding between PDA surface groups and cellulose chains in the hydrogels (Figure 2G). Characteristic peaks at 3346, 1147, 1017, and 896 cm^−1^ in ACH hydrogels corresponded to O–H stretching and C–O vibrations. The reduction in intensity of these peaks in ACHP hydrogels indicates not only covalent cross‐linking between cellulose hydroxyl groups and PDA surface groups but also the formation of hydrogen bonds (O–H⋯O/N). XPS analysis confirmed the presence of nitrogen from PDA on ACHP hydrogel surfaces (Figure 2H), with the nitrogen peak intensifying with increased PDA content. The high‐resolution C1s spectrum of ACHP (Figure 2I) exhibited peaks at 288.1 eV (O–C═O), 286.98 eV (O–C–O), 285.98 eV (C–O), 284.78 eV (C–N), and 284.1 eV (C–C), confirming the incorporation of PDA and the presence of multiple bonding types, including ether and carbon–nitrogen bonds. The N1s spectrum (Figure 2J) was deconvoluted into three components at 398.5 eV (–N═), 399.7 eV (–NH–), and 401.3 eV (–NH_2_), consistent with PDA self‐polymerization and covalent bonding with cellulose. These results collectively verify that the integration of PDA with ACH involves multiple interactions, including covalent bonding, hydrogen bonding, and possibly π–π stacking.

To assess the effects of stretching and different contents of PDA on the crystal form of cellulose hydrogels, the hydrogels were characterized via XRD (Figure 2K). The ACH and ACHP hydrogels showed peaks near 2*θ* = 12.3° and 20.6°, which corresponded to cellulose crystals in the (110) and (200) planes, respectively.^[^ ^40^ ^]^ After the piezoelectric hydrogels (ACH and ACHP) underwent stretching, the peak near 20.6° increased significantly, indicating that the stretching process ordered the fiber crystals inside the cellulose, and increased the crystallinity of the structure. Differential scanning calorimetry (DSC) was performed to further assess the effects of stretching and different contents of PDA on the crystallization of cellulose hydrogels (Figure 2L). Thermal analysis of the endothermic peaks corresponding to the melting process showed that pretensile treatment led to the absorption of significantly more energy, with fusion enthalpies of 99.89 J g^−1^ for ACH, suggesting a higher crystallinity content in the former. The fusion enthalpies after different PDA modifications increased relative to those of ACH, the peak melting temperature increased slightly, the peak intensity increased, and the peak intensity of ACHP reached a maximum, indicating that PDA promoted the functionalization of ACH and improved its thermal stability.

Next, small‐angle X‐ray scattering (SAXS) was performed to characterize the effects of stretching and different contents of PDA modification on the anisotropy of the hydrogels (Figure 2M; Figure S3D, Supporting Information). In the SAXS mode, CH had uniform strength rings, which reflected its isotropic structure. When the hydrogel was stretched to 120%, ACH formed a sharp, elongated equatorial streak, and the ring strength was anisotropic. Therefore, the ACH with a prestretching ratio of 120% was selected for all subsequent experiments. The core design lies in utilizing its highly anisotropic fibrous structure. SAXS results (Figure 2M) clearly demonstrated that prestretching induced a highly oriented fiber alignment, manifested as a distinct equatorial streak and a sharp intensity peak at the 90° azimuth. This anisotropic topological structure provides essential contact guidance for neural cells, effectively directing axonal extension, promoting orderly neuronal migration, and inhibiting disordered glial scar formation. This constitutes the fundamental requirement and core design rationale for achieving ordered neural regeneration following TBI^[^ ^10^ ^]^ Additionally, the modification of PDA did not significantly change the azimuth‐integral strength distribution curve (Figure 2M).

A prestretching strain of 120% (ACH) was selected for all subsequent experiments based on a comprehensive optimization of mechanical properties, piezoelectric output, and structural anisotropy. This specific strain balanced high tensile strength, enhanced piezoelectric, and well‐defined aligned nanostructure.

### 2.2 The Enhancement of Piezoelectric Performance, Stability, and its Mechanism of ACH by PDA

**Figure 3:**
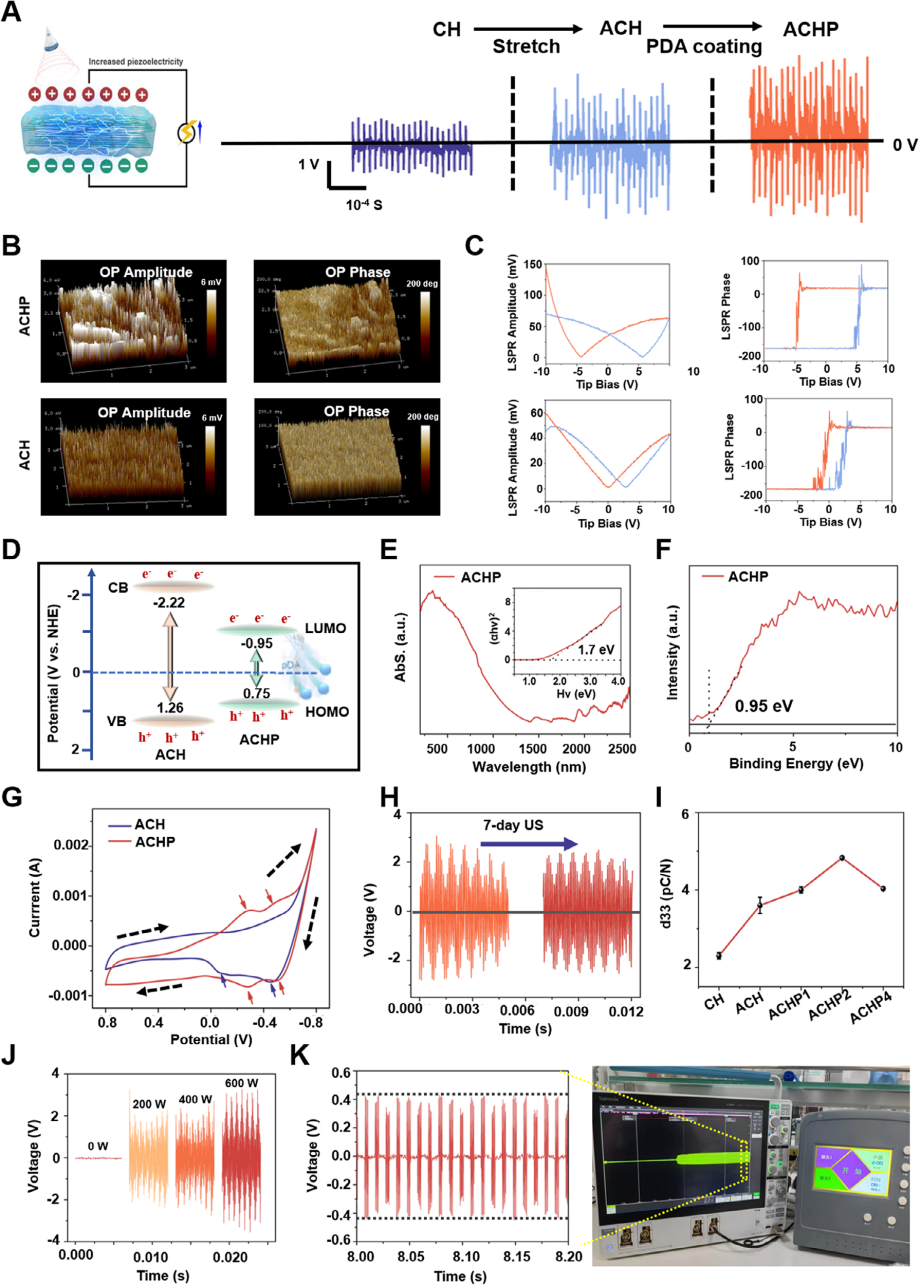
The enhancement of piezoelectric performance, stability, and its mechanism of ACH by PDA. A) Piezoelectric signals generated by different hydrogels under the same ultrasonic intensity. The voltage of the CH, ACH, and ACHP was found to be about 0.5, 1, and 1.5 V, respectively, confirming the ordered structure and the addition of PDA can improve the piezoelectric properties of hydrogels. B) PFM phase, and PFM amplitude images of ACH and ACHP. C) Phase potential hysteresis (Left) and butterfly‐shaped amplitude loop (Right) of ACH and ACHP when DC voltage ranged from −10 to 10 V, which proved that ACHP has good piezoelectric properties. D) Piezoelectric mechanism diagram of PDA enhanced cellulose hydrogel. E) UV diffuse reflection characterization of ACHP. F) VB‐XPS characterization of ACHP. G) CV curve characterization of ACH and ACHP. H) The change in piezoelectric properties of ACHP after 7 days of ultrasound treatment. I) The *d*_33_ values of cellulose hydrogels, the *d*_33_ constant increased significantly after stretching and the addition of PDA, showing the piezoelectric properties of hydrogel were significantly improved. J) Piezoelectric signal output of ACHP under different ultrasound stimulation. The piezoelectric signal increases with the increase of ultrasonic intensity, and when the ultrasonic intensity is 400 W, the output voltage is about 2.5 V. K) Actual picture of piezoelectric output and actual picture under 2 W cm^−2^ ultrasound in vivo.

The piezoelectric signal of the hydrogel was also tested using an oscilloscope and an ultrasonic instrument to evaluate its piezoelectric properties (**Figure** **3**A). The voltage of the ordered ACH was 1 V, which was significantly greater than that of the disordered CH (0.5 V). These results indicated that the ordered structure significantly improved the piezoelectric properties of the hydrogel. Additionally, the modification of PDA significantly improved the piezoelectric properties of ACH, and the maximum voltage of ACHP was 1.5 V. These findings further indicate that the ordered structure and the addition of PDA can improve the piezoelectric properties of hydrogels.

Piezoelectric force microscopy (PFM) is a useful scanning probe technique that can directly measure piezoelectricity. The first piezo‐characterization of cellulose solutions showed a typical butterfly‐shaped amplitude curve with a bias voltage ranging from −10 to +10 V, indicating a constantly changing strain due to the applied electric fields. On the phase diagram, the presence of a 180‐phase switch in the local piezoelectric hysteresis loop of the cellulose solution indicated piezoelectric properties (Figure S1B, Supporting Information). In PDA‐modified nanocellulose hydrogels, the nanocellulose acts as an energy source, and the PDA coating acts as a binder and stress enhancer at the two‐phase interface. This results in a stronger electromechanical coupling effect, and thus, a greater piezoelectric response. The PFM phase maps, and the corresponding amplitudes of ACH and ACHP (Figure 3B). Compared to the piezoelectric amplitude image of ACH, the piezoelectric amplitude image of ACHP had a stronger phase contrast. The ferroelectric properties of the anisotropic cellulose hydrogels were confirmed by the local field polarization hysteresis of the PFM phase and amplitude loops. When a bias switch was applied, all hydrogels exhibited a typical butterfly‐shaped amplitude curve and a significant hysteresis corresponding to a 180° phase change (Figure 3C; Figure S7, Supporting Information). ACHP had the smoothest curve and the largest hysteretic area, which indicated that the ferroelectric properties of ACHP were more stable.

To uncover the mechanism by which stretching enhance piezoelectric properties, we conducted peak fitting on XRD patterns (Figure S6, Supporting Information). The results revealed that the β‐phase content of ACH was nearly threefolds that of CH, while the β‐phase content of ACHP remained largely unchanged. This indicates that stretching effectively induces self‐polarized cellulose piezoelectric nanofibers with a high piezoelectric β‐phase, significantly enhancing charge separation efficiency.

We hypothesize that the mechanism by which PDA enhances piezoelectricity involves two primary aspects: First, the PDA coating can form heterostructures with piezoelectric nanomaterials, which facilitate electron migration and improve the charge separation and conduction rates of the piezoelectric material. Through ultraviolet diffuse reflectance spectroscopy characterization (Figure 3E; Figure S6A, Supporting Information), we calculated that the electronic bandgap of ACHP (1.7 eV) is smaller than that of ACH (3.48 eV, as shown in Figure S8A in the Supporting Information), making PDA a favorable medium for charge transport during ultrasonic stimulation, thus promoting electron migration. Notably, based on the VB‐XPS spectra (Figure 3F; Figure S8B, Supporting Information), we determined the maximum valence band (VB) and minimum conduction band (CB) values of ACHP to be 0.75 and −0.95 eV, respectively, while those of ACH are 1.26 and −2.22 eV. These results indicate that when the piezoelectric material is stimulated by ultrasound, it generates charges, and the PDA coating acts as an effective charge separation layer, promoting the separation of electron–hole pairs, thereby enhancing the charge separation efficiency of the piezoelectric material. Additionally, the PDA coating, acting as an “electron extractor” (a channel for electron transport), accelerates electron migration, thus increasing the charge conduction rate of the piezoelectric material (Figure 3D). Second, the capacitive effect of PDA primarily stems from its rich redox‐active groups, such as amines and phenolic hydroxyls, which can participate in electrochemical reactions and facilitate electron transfer. This effect can improve the piezoelectric performance of the material. As shown in Figure 3G, the cyclic voltammetry (CV) behavior of ACH and ACHP indicates a significant improvement in the electrochemical performance of ACHP. The CV curve of ACHP exhibits two pairs of redox peaks, demonstrating its capacitive behavior in the electrochemical process. Moreover, the integral area of the CV curve of ACHP was notably larger than that of ACH. These results suggest that PDA modification increases the capacitive effect of ACH, promotes electron transfer, and enhances the material's piezoelectric performance.

We also observed through CV curves that ACH has only two reduction peaks without corresponding oxidation peaks, indicating that pure ACH, once it loses electrons, cannot regain them and is unstable. In contrast, ACHP has two pairs of redox peaks, indicating that ACHP can regain electrons after losing them, which proves the reason for the more stable piezoelectric output after PDA modification. We observed that after 7 d of ultrasound treatment, the piezoelectric properties of ACHP only slightly decreased (Figure 3H), which further indicates that PDA modification increases the stability of piezoelectric output.

To further assess the piezoelectric properties of different hydrogels, they were first freeze‐dried, and subsequently combined with the upper and lower electrodes to determine their d_33_ values. ACH had a *d*_33_ value of 3.7 pC N^−1^, which was greater than the *d*_33_ value of CH (2.3 pC N^−1^) (Figure 3I). PDA‐modified ACHP also had a high *d*_33_ value; the maximum *d*_33_ value of ACHP was 4.8 pC N^−1^. These results showed that the anisotropic structure and the modification of PDA significantly improved the piezoelectric properties of the hydrogel.

Next, we evaluated the piezoelectric response of ACHP in aqueous solution under ultrasound stimulation at a fixed frequency of 20 kHz and varying intensities (0, 200, 400, and 600 W). No electrical signal was detected in the absence of ultrasound (Figure 3J). When ultrasound was applied, a distinct electrical signal was recorded, and 200, 400, and 600 W ultrasound produced wireless electrical stimuli in the 2–4 V range. The wireless electrical signal increased consistently with ultrasound intensity, demonstrating a controllable piezoelectric response suitable for ultrasonic energy harvesting applications. ACHP and electrodes were implanted under the skull of rats (Figure S9, Supporting Information), and a voltage of about 0.4 V was output after ultrasound treatment of 2 W cm^−2^ and 1 MHz (Figure 3K).

### 2.3 Cell Adhesion and Biocompatibility of ACHP In Vitro

Given that surface roughness is a critical factor influencing cell adhesion, we subsequently employed atomic force microscopy (AFM) to analyze the roughness of the samples. As depicted in Figure S10 (Supporting Information), after stretching, the surface roughness of ACH increased compared to CH. Moreover, the modification of the hydrogel surface with PDA resulted in a significant enhancement in the sample's roughness. This could potentially have a positive impact on cell adhesion. We further performed phalloidin staining on neural stem cells (NSCs) cultured for two days, and the results are shown in Figure S11 in the Supporting Information. A small number of NSCs were observed on CH, clustered together without the spread of lamellipodia. In contrast, a noticeable increase in cell adhesion was observed on ACH, with cells showing slight elongation and spreading. Most importantly, on ACHP, the cells exhibited enhanced spreading, with long and stable edges; the spread area of the cells increased, indicating that PDA improved the material's roughness, inducing a tight contraction between the cells and the substrate. ACHP exhibits stable properties under physiological conditions (PBS and collagenase), yet its primary components‐nanocellulose and polydopamine‐remain biodegradable. In vitro cellulase degradation assays (25 U mL^−1^) demonstrated that ACHP achieved a degradation rate exceeding 90% within 7 d, indicating near‐complete degradation. These results confirm its favorable environmental compatibility and controllable degradation behavior, with metabolites that are non‐toxic and harmless to both organisms and the environment (Figure S12, Supporting Information).

We evaluated ACHP hydrogel biocompatibility at different ultrasound intensities using live/dead staining (Figure S13, Supporting Information). Without ultrasound, ACHP‐supported NSCs showed 97.9% viability, surpassing TCP's 90.9%, likely due to the hydrogel's flexibility and PDA‐induced biocompatibility. NSCs viability on ACHP was maintained at 0, 200, and 400 W ultrasound, with a notable drop at 600 W, suggesting an ultrasound intensity threshold. We selected 400 W for further studies to balance cell response and hydrogel performance, as it provided sufficient electrical output (≈2.5 V) while maintaining high cell viability (>90%).

### 2.4 Antioxidant and Anti‐Inflammatory Properties of Immuno‐Piezoelectric Transducers

**Figure 4:**
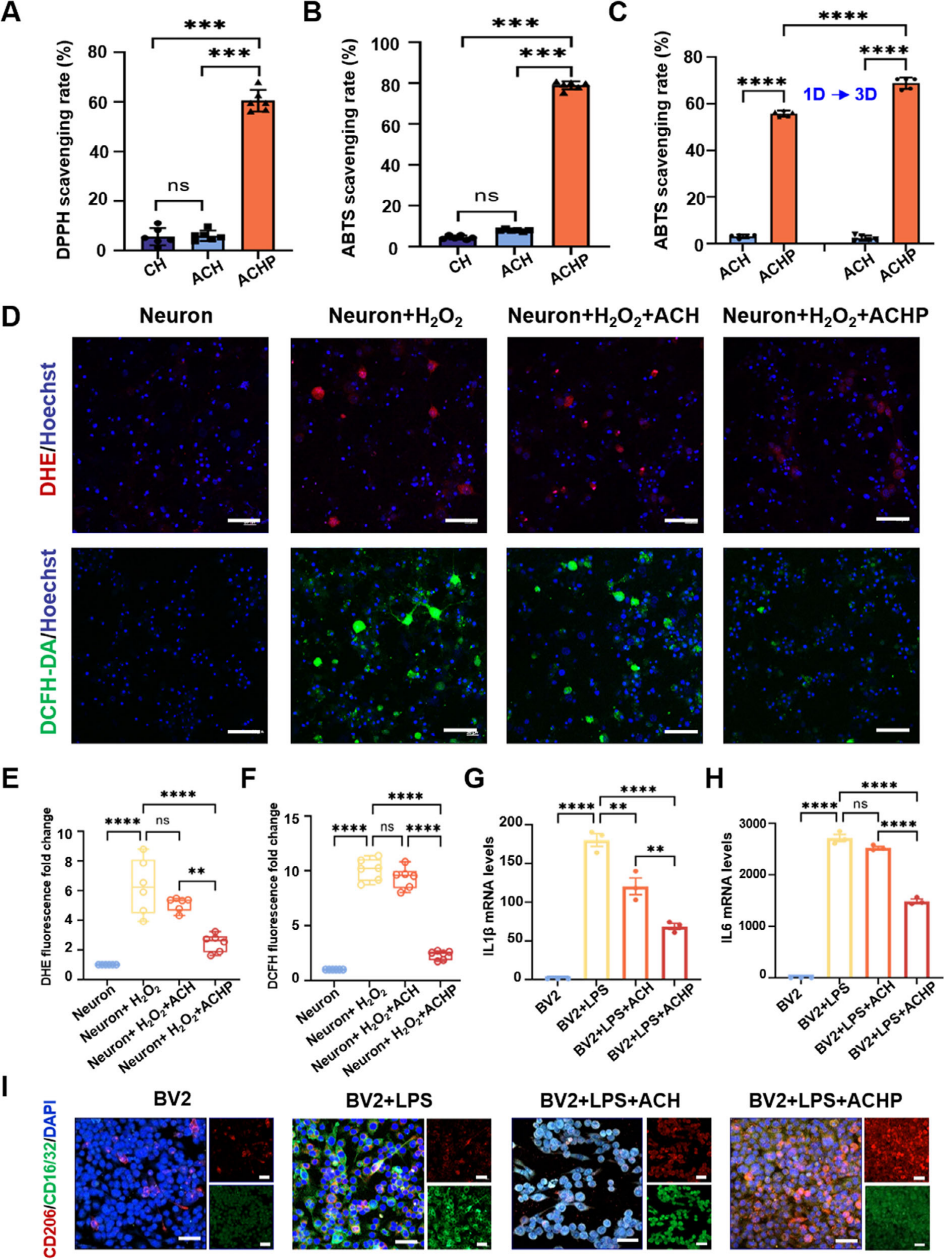
In vitro anti‐oxidative and anti‐inflammatory ability of dual‐mode transducer. Antioxidant properties of different cellulose hydrogels, determined by the A) DPPH method and the B) ABTS method, confirming PDA‐modified hydrogels have excellent antioxidant activity, when the hydrogel concentration was 10 mg mL^−1^, the clearance rate of ROS was 80%. C) ACHP was treated with ultrasound for 1 and 3 d, respectively, and the antioxidant properties of extraction were determined by the ABTS method. D) Neurons under oxidative stress: Representative immunofluorescence images of neurons treated with hydrogen peroxide (H_2_O_2_) to induce oxidative stress. DHE (red) staining indicates intracellular reactive oxygen species (ROS) levels. DCFH‐DA (green) staining reflects general oxidative stress levels. *n* = 3. Scale bar, 50 µm. Quantitative detection of E) DHE and F) DCFH‐DA fluorescence intensity. qRT‐PCR assay to detect the mRNA levels of inflammatory cytokines G) IL1β and H) IL6 in BV2 cells cultured on hydrogels induced by LPS. *P < 0.05, **P < 0.01, ***P < 0.001, ****P < 0.0001. I) BV2 microglial polarization: Representative immunofluorescence images of BV2 microglial cells under lipopolysaccharide (LPS)‐induced inflammatory conditions. CD206 (red) indicates M2 anti‐inflammatory microglial polarization. CD16/32 (green) indicates M1 pro‐inflammatory microglial polarization. DAPI (blue) marks the cell nuclei. *n* = 3. Scale bar, 50 µm.

To test the hypothesis that PDA endows hydrogels with the ability to clear ROS from brain injury under high oxidative stress, the antioxidant potential of different cellulose hydrogels was studied by the 1‐diphenyl‐2‐pyridylhydrazyl radical (DPPH) method (**Figure** **4**A) and the 2, 2‐nitro‐bis (3‐ethylbenzothiazole‐6‐sulfonic acid (ABTS) method (Figure 4B). PDA‐modified cellulose hydrogels had higher scavenging capacities than CH and ACH. When the hydrogel concentration was 10 mg mL^−1^, the clearance rate of ROS was 80%. The abundant antioxidant groups in PDA (such as phenolic hydroxyl groups) are responsible for removing ROS.^[^ ^41^ ^]^ Therefore, PDA‐modified hydrogels have excellent antioxidant activity and are conducive to the removal of ROS in brain‐damaged areas.

To further understand the antioxidant capacity of PDA‐modified cellulose hydrogels under ultrasound stimulation, the cellulose hydrogels (ACH and ACHP) were treated with ultrasound for one or three days. The results of the ABTS assay showed that the antioxidant capacity of ACHP was significantly greater than that of ACH without ultrasound stimulation (Figure 4C). For ACHP, the antioxidant capacity after ultrasound stimulation was significantly greater than that without ultrasound stimulation. The results showed that PDA was the main antioxidant component in cellulose hydrogels, and the content of PDA in the extraction solution increased after ultrasound stimulation which provided cellulose hydrogels with the opportunity to improve the brain injury environment.

We have detected the antioxidant and anti‐inflammatory functions of PDA‐modified piezoelectric hydrogel itself, but the real function needs to be verified at the cellular level, so we designed and conducted the following experiments.

To verify the antioxidant function, we inoculated the identified rat primary NSCs (Figure S14, Supporting Information) on coverslip, ACH and ACHP materials respectively. After 7 d of differentiation, 200 × 10^−6^ m H_2_O_2_ was added for 3 d, and then superoxide anion DHE and reactive oxygen ROS fluorescent probe DCFH‐DA were identified respectively. The results showed (Figure 4D) that compared with the Neuron group, the Neuron+H_2_O_2_ group highly expressed DHE and DCFH‐DA, indicating that H_2_O_2_ can induce neurons to be in an oxidative state. Compared with the Neuron+H_2_O_2_ group, the DHE and DCFH‐DA in the Neuron+H_2_O_2_+ACH group did not decrease, and the DHE (Figure 4E) and DCFH‐DA (Figure 4F) in the Neuron+H_2_O_2_+ACHP group decreased significantly, indicating that the PDA‐modified piezoelectric hydrogel machine ACHP has antioxidant function.

Brain injury also activates microglia, prompting them to adopt an inflammatory phenotype. This triggers the overproduction of pro‐inflammatory cytokines, including interleukin‐1β (IL‐1β) and IL‐6. Consequently, we evaluated the in vitro anti‐inflammatory capabilities of ACH and ACHP through co‐culture with microglia (BV2). Lipopolysaccharide (LPS) is a potent inflammatory response stimulant that can induce a pro‐inflammatory response in BV2 cells. The expression of pro‐inflammatory markers (IL‐1β and IL‐6) mRNA was significantly downregulated after treatment with ACHP hydrogel, as determined by real‐time quantitative polymerase chain reaction (qRT‐PCR) (Figure 4G,H). Immunofluorescence staining further demonstrated the regulatory effect of ACHP on microglial polarization (Figure 4I; Figure S15, Supporting Information). Compared to the BV2 group, LPS stimulation significantly increased the expression of the pro‐inflammatory marker CD16/32, confirming successful induction of the M1 phenotype. Relative to the LPS‐induced group, the ACH group showed a slight increase in the M2 marker CD206 and a slight decrease in CD16/32. In contrast, the ACHP groups exhibited a significant increase in CD206 expression and a notable reduction in CD16/32 compared to the ACH group.

To specifically determine the role of PDA itself in driving M2 polarization of microglia, a key control group was included: BV2 cells cultured on glass coverslips coated with in situ polymerized PDA (TPDA), under the same dopamine concentration conditions (2 mg mL^−1^). Statistical results (Figure S15, Supporting Information) indicated no significant difference in promoting CD206⁺ M2 microglia between the TPDA and ACHP groups, while both groups showed significantly higher proportions of CD206⁺ cells compared to the ACH group. These findings confirm that PDA itself is the decisive factor driving the anti‐inflammatory polarization of microglia.

### 2.5 Piezoelectric Transducer Promotes the Differentiation and Development of NSCs by PI3K‐Akt Signaling Pathway

**Figure 5:**
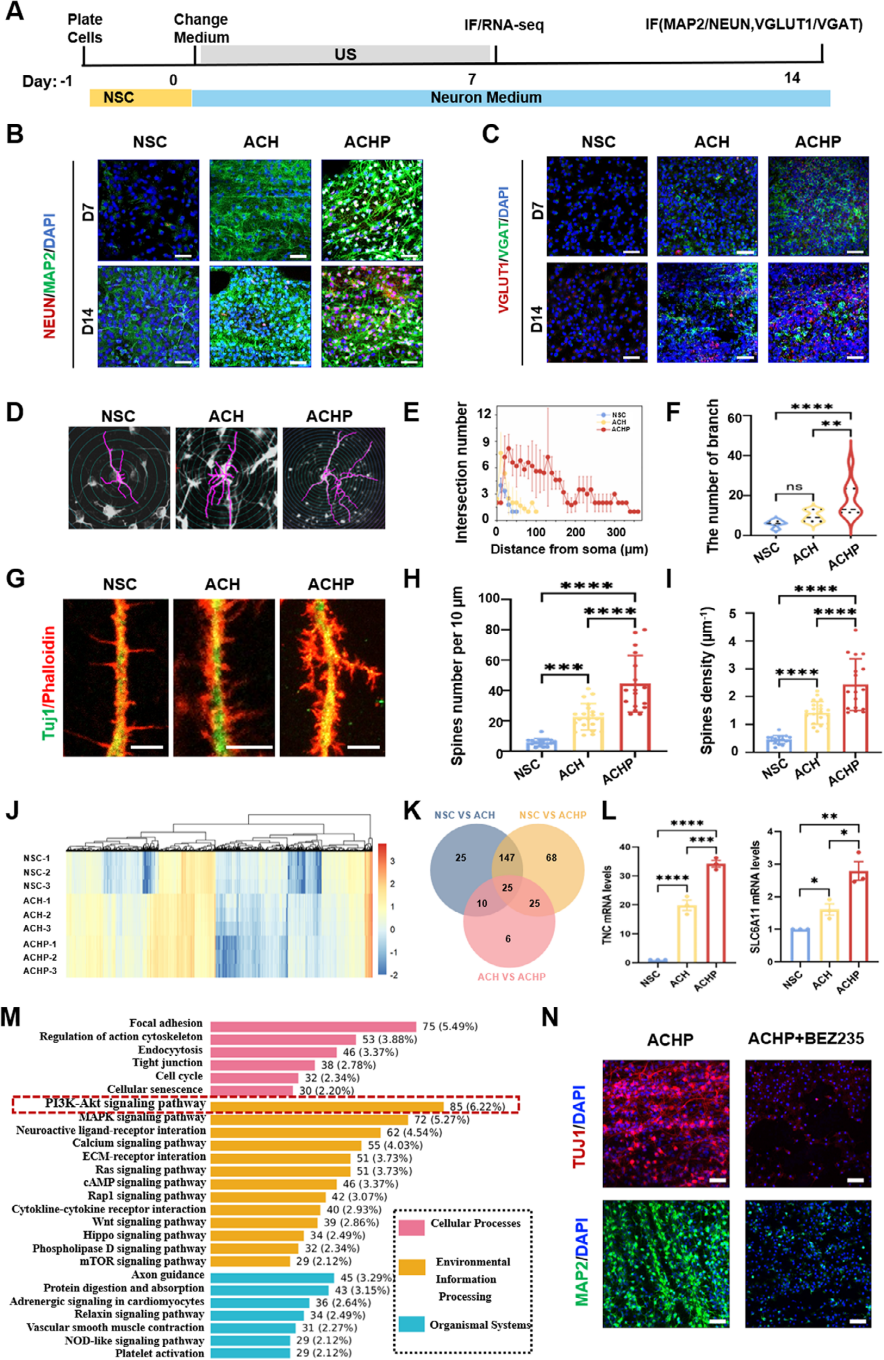
Study on differentiation, development and mechanism based on RNA sequencing of NSCs. A) Experimental Design: Timeline of the neuronal differentiation experiment. NSCs were plated on Day ‐1, the medium was changed to neuron medium on Day 0, and ultrasound (ultrasound) stimulation was applied until Day 7. Samples were analyzed via immunofluorescence (IF), RNA sequencing at specified time points (Day 7 and Day 14). B) Neuronal Marker Expression: Immunofluorescence images of neuronal differentiation at Day 7 (D7) and Day 14 (D14) under different treatments: Neun (red): Marker of mature neurons: MAP2 (green): Marker of neuronal dendrites. C) VGLUT1 (red): Marker of excitatory synapses. VGAT (green): Marker of inhibitory synapses. DAPI (blue): Nuclear counterstain. *n* = 3. Scale bars, 50 µm. D) Neuronal cytoskeleton reconstruction of NSCs cultured on NSC, ACH, and ACHP for 7 d using the Neurolucida software. E) Sholl analysis plots comparing dendritic complexity and F) branch number of neurons derived from NSCs differentiation on NSC, ACH, and ACHP for 7 d under wireless ultrasound stimulation at least five neurons per group. G) Representative images of dendritic spines stained by neuronal markers Tuj1 (green) and skeleton staining phalloidin (red) after 7 d of culture on NSC, ACH, and ACHP. scale bar: 5 µm. (H and I) Statistical analysis of dendrite spine density, spine density of neurons derived from NSCs show significant higher spine density when cultivated on PA under wireless ultrasound stimulation (*n* = 3). J) K‐means cluster analysis of differentially expressed genes among NSCs cultured for 7 d on NSCti‵ACH and ACHP with and without wireless electrical stimulation, respectively; K) The Venn map of NSC, ACH and ACHP differential genes in KEGG pathway was selected. L) Selected genes related to the neural differentiation that are upregulated in ACHP. Their respective fold changes measured by RNA‐seq are as shown. *P < 0.05, **P < 0.01, ***P < 0.001, ****P < 0.0001. M) The KEGG pathway classification of differentially expressed genes between NSCs cultured with wireless electrical stimulation on NSC and ACHP for 7 d. N) The immunofluorescence staining of early neuron marker Tuj1(red), and mature neuron marker MAP2 (green) was performed after treatment with BEZ235; scale bar: 50 µm. All data are expressed as the mean ±standard deviation (SD; *n* = 3).

To assess the effect of immuno‐piezoelectric transducer on NSCs differentiation into neurons, we used immunofluorescence and RNA‐seq to analyze neuron‐related gene expression. On Day‐1, NSCs were seeded onto coverslips with ACH or ACHP. On Day 0, the NSCs proliferation medium was replaced with differentiation medium, and ultrasound stimulation was applied twice daily for 7 d (**Figure** **5**A).

Immunofluorescence staining for MAP2 and Neun (Figure 5B) showed increased MAP2 expression in the ACH group compared to the NSC group, with the ACHP group showing the highest expression. On Day 14, Neun expression was significantly higher in the ACHP group compared to both the NSC and ACH groups. Next, we evaluated neuronal subtypes using glutamatergic neuron marker VGLUT1 and GABAergic neuron marker VGAT (Figure 5C). Compared with the NSC group, the ACH group showed an increase in VGAT expression on both Day 7 and Day 14, and the ACHP group showed an even greater increase. The ACHP group also showed a significant increase in VGLUT1 expression compared to the ACH group. On Day 14, compared to Day 7, the ACHP group exhibited a significant increase in VGLUT1 and a decrease in VGAT. These results suggest that immuno‐piezoelectric transducer significantly promote the differentiation of neural stem cells into Neun^+^ neurons and favor the differentiation of NSCs into VGLUT1^+^ glutamatergic neurons.

To evaluate the effects of the immuno‐piezoelectric transducer on the development of NSC‐derived neurons, we stained neurons from each group with the neuron‐specific marker Tuj1 and analyzed synaptic complexity using Neurological software^[^ ^42^, ^43^ ^]^ (Figure 5D). Sholl analysis (Figure 5E) revealed the NSCs differentiated on the ACHP exhibited multipolar features, including axon elongation (≈360 µm) and an increase in axon dendrites. In contrast, the axon lengths were ≈100 µm for ACH and 50 µm for NSC. Compared to the other three groups, neurons in the ACHP group displayed longer neurites and more dendrites, with a maximum of 37 branches (compared to fewer than 15 branches in the other groups) (Figure 5F).

Dendritic spine density was analyzed to evaluate the synaptic plasticity of NSC‐derived neurons on the wireless piezoelectric transducers. Dendritic spine density is a key indicator of synaptic plasticity. Slender and filamentous spines are considered immature, while short, thick, or mushroom‐shaped spines are considered mature.^[^ ^44^ ^]^ NSC‐derived neurons were further evaluated using F‐actin immunostaining and Tuj1 labeling to investigate synaptic plasticity. The results showed that the NSC‐derived neurons on the ACH exhibited more F‐actin‐positive dendritic spines compared to the NSC, with an even greater number of spines observed in the ACHP group (Figure 5G). Further analysis revealed that the dendritic spine density in the ACH group (1.50 ± 0.34) was significantly greater than that in the NSC group (0.44 ± 0.12) (Figure 5H). Notably, the density of dendritic spines in the ACHP group (2.43 ± 0.9) was nearly double that in the ACH group. The ACHP group exhibited significantly more spines per 10 µm compared to the other three groups (Figure 5I). These results indicated that the immuno‐piezoelectric transducer provide a conducive microenvironment for neurite growth and effectively promote the development and maturation of neurite, enhancing synaptic plasticity.

RNA sequencing was performed on the NSC, ACH, and ACHP groups over 7 d to explore the molecular mechanisms underlying neural differentiation (Figure 5J–N; Figure S16, Supporting Information). K‐means clustering analysis (Figure 5J) confirmed high uniformity among triplicate samples across NSC, ACH, and ACHP groups. The Venn diagram analysis identified 25 differentially expressed genes (DEGs) between the NSC and ACH groups, and only 6 DEGs between the ACH and ACHP groups (Figure 5K), suggesting that wireless electrical stimulation as the dominant driver of transcriptional changes in NSCs on piezoelectric hydrogel. Additionally, upregulation of neural differentiation‐related genes (*TNC, CACNA1B, Gabbr2, CALB1*) was observed, demonstrating electrical stimulation precisely regulates neurogenic programs (Figure 5L; Figure S16B, Supporting Information). KEGG pathway analysis of DEGs (Figure 5M; Figure S16A, Supporting Information) revealed significant enrichment in neural processes including “PI3K‐Akt signaling pathway”, “Neuroactive ligand‐receptor interactions”, “Calcium signaling”, and “Axon guidance”. Crucially, as electrical stimulation constitutes a physical cue, we focused on the “Environmental Information Processing” category, where the PI3K‐Akt pathway showed the most prominent enrichment, highlighting its pivotal role in transducing electrical signals into pro‐differentiation biochemical responses. To test the necessity of PI3K‐Akt signal transduction, we treated with BEZ235 (an PI3K‐Akt/mTOR dual inhibitor). The results showed that BEZ235 significantly inhibited the expression of the early neuronal marker Tuj1 and the mature neuronal marker MAP2 (Figure 5N), which confirms the important role of the PI3K‐Akt/mTOR axis in the pro‐differentiation effect of piezoelectric‐mediated wireless electronic stimulation. After treatment with P529 (Akt inhibitor), the expression of MAP2 was specifically reduced (Figure S16C, Supporting Information), further verifying the key function of Akt in promoting neuronal maturation under wireless electronic stimulation.

These findings demonstrate that immuno‐piezoelectric transducer, particularly ACHP, significantly enhance the differentiation and development of NSCs into functional neurons, promoting neurite outgrowth, synaptic plasticity by activating PI3K‐Akt. This approach provides a promising strategy for neural tissue engineering and regenerative medicine.

### 2.6 Immuno‐Piezoelectric Transducer Inhibits Inflammation and Promotes NSC Differentiation In Vivo

**Figure 6:**
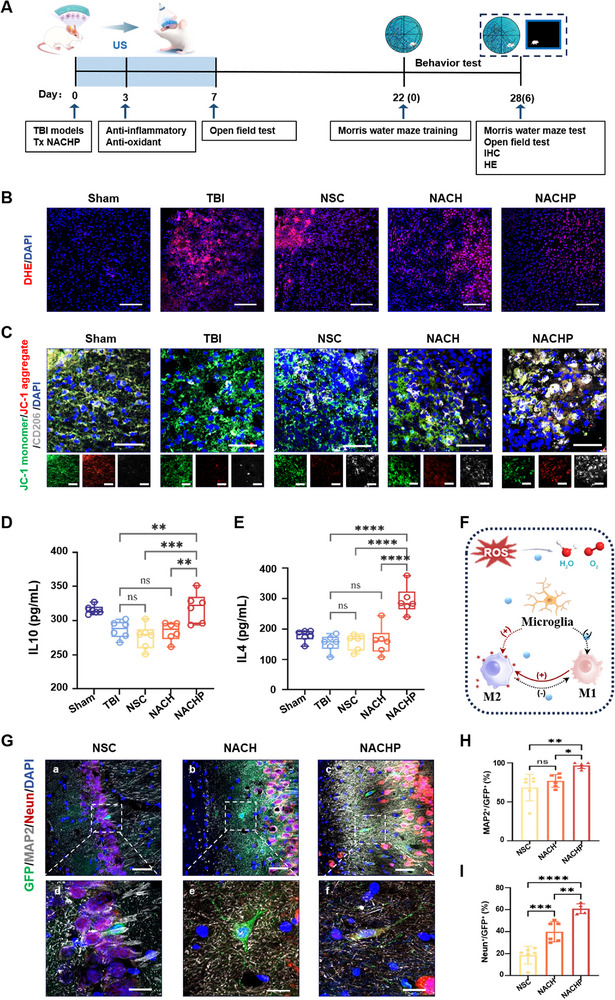
Immuno‐piezoelectric transducer improved the immune microenvironment of early TBI injury and promoted the differentiation of exogenous NSCs into neurons in the junction area of injury. A) Experimental Timeline: Schematic representation of the experimental design. Treatments (TBI induction, NSCs transplantation, NACH, and NACHP) and assessments (anti‐inflammatory tests, open field test, Morris water maze, and histological analyses) were conducted at the specified time points. ultrasound stimulation was applied from Day 1 to Day 7. B) Oxidative Stress Assessment (DHE Staining): Representative images of DHE staining showing oxidative stress levels across treatment groups: Sham, TBI, NSC, NACH, and NACHP. *n* = 3. Scale bar, 100 µm. C) Immunofluorescence images showing the expression of JC‐1 (green, monomer; red, aggregate) and CD206 (white) in coronal brain sections. Treatment groups include Sham, TBI, NSC, NACH, and NACHP. Insets highlight localized areas of inflammation and glial activation. *n* = 6, Scale bar, 50 µm. Concentration of D) anti‐inflammatory cytokine IL‐10 and E) anti‐inflammatory cytokine IL‐4 measured via ELISA in brain tissue lysates from different treatment groups, *n* = 6. F) Schematic of improving microenvironment by wireless immunoregulatory piezoelectric transducers. G) Neuronal Regeneration Assessment: Representative images of neuronal markers MAP2 (green) and Neun (red) in the peri‐injury region across treatment groups. Enlarged views show detailed neuronal recovery. *n* = 6. a–c) scale bar: 50 µm; d–f) scale bar: 20 µm. H) MAP2^+^/GFP^+^ and I) Neun^+^/GFP^+^ statistical analysis on different groups for 28 d (*n* = 6). * *p* < 0.05, ** *p* < 0.01, *** *p* < 0.001, and **** *p* < 0.0001.

Prior to in vivo implantation, ACHP was evaluated for biocompatibility by subcutaneous and intracerebral implantation in healthy SD rats. H&E and CD86 staining revealed no significant inflammatory response at day3, day7, and day14 post‐implantation, confirming its excellent biocompatibility for subsequent experimental use (Figure S17, Supporting Information). The in vivo efficacy of immuno‐piezoelectric transducer in treating traumatic brain injury (TBI) was evaluated using brain injury model in SD rats. The mNSS is a combination of motor, sensory, reflex, and balance tests used to assess the severity of trauma.^[^ ^45^, ^46^ ^]^ A higher score indicates a more severe injury. Rats with moderate‐to‐severe TBI (mNSS score > 8 at 24 h post‐injury) were selected for subsequent experiments to ensure injury consistency. Multi‐modal MRI (T2, DWI, ADC) confirmed uniform lesion size and location across animals (Figure S18, Supporting Information). Rats were divided into five experimental groups: Sham (no TBI), TBI (PBS injection), NSC (GFP‐NSC injection, Figure S19, Supporting Information), NACH (NSC+ACH hydrogel), and NACHP (NSC+PDA‐modified ACH hydrogel). The inflammatory response and neuroinflammatory cell influx peaked within 3 d after TBI, and 28 d after TBI is another critical time point for the transplanted neural stem cells to differentiate into mature neurons. Therefore, Rats were euthanized at Days 3 and 28 for analysis of brain tissue (**Figure** **6**A).

First, utilizing a chemical cross‐linking method, we conjugated the Cy5.5 fluorophore to the PDA layer on the surface of ACHP nanoparticles and implanted them into a TBI rat model (Figure S20A,B, Supporting Information). 3 d postimplantation, red fluorescent signals detected in brain slices confirmed that a portion of the PDA had dissociated and successfully penetrated the brain tissue (Figure S20C, Supporting Information). Next, in terms of regulating the early immune microenvironment regulation of TBI, the ROS levels in the Sham, TBI, NSC, NACH, and NACHP group were compared during the visualization of the injured area with DHE staining for 3 d, as shown in Figure 6B. Obvious DHE signals were detected after 3 d of TBI, indicating the existence of a large amount of accumulated ROS in the injured area. The level of ROS in NSC and NACH group did not decrease, and after NACHP treatment, the lowest fluorescence intensity of DHE in the damaged area could be observed, indicating that it had the strongest ability to remove ROS in the damaged area.

Microglia are highly plastic cells with phagocytic activity. In this study, microglia were activated after TBI,^[^ ^47^ ^]^ and two different phenotypes of activated microglia were recorded, which included classically activated pro‐inflammatory M1 microglia and selectively activated anti‐inflammatory M2 microglia. They play opposite roles in the recovery of brain function.^[^ ^48^ ^]^ M1 microglia can aggravate brain damage by releasing destructive pro‐inflammatory cytokines. In contrast, M2 microglia can alleviate local neuroinflammation and promote brain injury repair. Therefore, the regulation of microglia plays an important role in brain injury repair. First, we examined the distribution of M2‐type microglia in the injured area 3 d after TBI. At 3 d after TBI, immunofluorescence staining with the anti‐inflammatory marker CD206 (Figure 6C) was used to evaluate the transformation of microglia in the injured area after different treatments. In the NACHP group, microglia were polarized into a neuroprotective phenotype, showing a significant increase in CD206 positive cell. The anti‐inflammatory nanogenerator caused the polarization of microglia to neuroprotective M2 microglia. Additionally, mitochondria are vital energy carriers for maintaining neural function, and we further investigated the protective effect of NACHP on mitochondria under conditions of injury. Using the JC‐1 staining kit to assess the impact of NACHP on the mitochondrial membrane potential in M2 microglia, we found that in the TBI environment, there was a low ratio of JC‐1 aggregates (red) to monomers (green), indicating a decrease in MMP in M2 cells under ROS damage, which suggests a decline in mitochondrial stability and abnormal energy metabolism in M2 microglia. However, after treatment with NSC, NACH, and NACHP, the membrane potential in the NSC and NACH groups was like that of the TBI group, while the NACHP group showed an enhanced mitochondrial membrane potential (with an increase in JC‐1 aggregates). The relevant research results indicate that immuno‐piezoelectric transducer promote the transition to M2‐type microglia and enhance their activity. Subsequently, on the third day, the tissue supernatant of the injury site was completely extracted, and the expression levels of anti‐inflammatory cytokines IL‐4 and IL‐10 involved in the inflammatory response were detected by ELISA. The results showed (Figure 6D,E) that the expression of these cytokines was significantly upregulated after NACHP hydrogel treatment compared with the NACH group, indicating that NACHP exerted an anti‐inflammatory effect (Figure 6F).

The activation of glial cells (such as microglia and astrocytes) caused by the post‐traumatic inflammatory cascade increased the secretion of proinflammatory cytokines and chemokines and led to the pathological consequences of secondary neuronal injury. Therefore, immunofluorescence double staining of ionized calcium‐binding adaptor protein 1 molecule (Iba1) and glial fibrillary acid protein (GFAP) was performed in brain sections to highlight the long‐term presence of activated microglia and reactive astrocytes. As shown in Figure S21 (Supporting Information), the expression levels of Iba1 and GFAP in the affected and contralateral hemispheres of the TBI group were higher than those in the Sham group, indicating that the TBI model activated microglia and astrocytes, leading to the diffusion of ROS and inflammatory cytokines, resulting in an overall inflammatory response in the brain tissue. After 3 d of treatment of TBI rats with NACHP hydrogel, the number of Iba1^+^ microglia and GFAP^+^ astrocytes were significantly reduced. Magnified images of the injured area of the cerebral cortex showed that the processes of microglia and astrocytes in the NACHP‐treated group were less than those in the TBI group, further indicating that NACHP inhibited the activation of microglia and astrocytes. These results from the expression of neuroinflammatory proteins/genes reflect that NACHP plays a fundamental role in reducing the overall proinflammatory response. This response can prevent long‐term tissue damage after injury caused by neuroinflammation and neuronal atrophy after TBI.

MAP2 and Neun staining were used to assess NSCs differentiation into neurons 28 d after injury (Figure 6G; Figure S22, Supporting Information). The NACHP group showed significantly higher percentages of MAP2^+^ and Neun^+^ neurons compared to the NSC and NACH groups (Figure 6H,I). These results indicate that NACHP hydrogel not only reduces neuroinflammation but also promotes the survival and differentiation of transplanted NSCs into mature neurons.

In summary, the immuno‐piezoelectric transducer significantly modulates the immune microenvironment, enhance NSCs survival, and promote their differentiation into functional neurons, making it a promising therapeutic strategy for TBI.

### 2.7 Immuno‐Piezoelectric Transducer with NSCs Improves TBI Structure, Function, and Behavior

**Figure 7:**
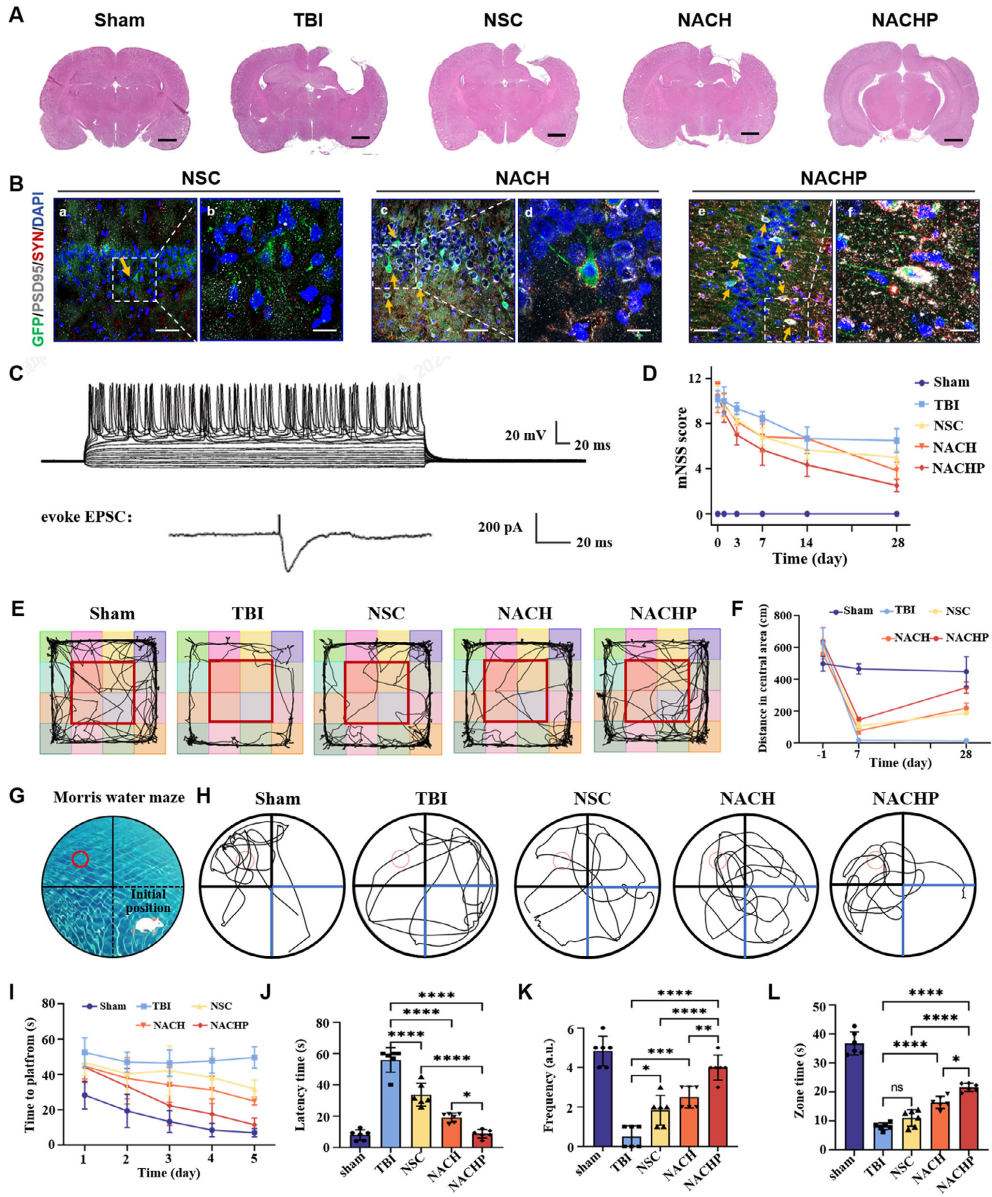
NACHP promotes structural, functional and behavioral recovery of TBI rat. A) Histological Analysis of Brain Sections: Representative H&E‐stained coronal brain sections from different treatment groups (Sham, TBI, NSC, NACH, NACHP). The images demonstrate the reduction in lesion size and tissue damage in the NACH and NACHP groups compared to other treatments, scale bar: 1000 µm. B) The presynaptic marker SYN (red) and the postsynaptic marker PSD95 (white) were stained by immunofluorescence after 28 d. Treatment groups: Sham, TBI, NSC, NACH, NACHP. *n* = 6. a,c,e) scale bar: 50 µm; b,d,f) scale bar: 20 µm. C) Electrophysiological characterization and synaptic connection of transplanted cells after 28 d of TBI. D) Neurological Deficit Scores: Neurological function was evaluated over 14 d post‐TBI. Scores were compared among Sham, TBI, NSC, NACH, and NACHP groups, demonstrating a significant improvement in the NACHP group. *n* = 6. E) 28‐d post‐TBI field map F) Premolding, 7‐d post‐TBI field tests, and 28‐day post‐TBI field tests: Statistical maps of travel through the central area. G) Morris Water Maze (MWM) Experimental Setup: Schematic representation of the MWM test, with a hidden platform located in the target quadrant. H) MWM Swimming Trajectories: Representative swimming paths of mice from each treatment group during the MWM test. NACHP‐treated mice showed improved spatial learning and memory, indicated by shorter paths and more direct routes to the hidden platform. *n* = 6. I) Time to platform significant declined through the platform quadrant on NACHP group within five days, showing the learning ability was significantly improved after treatment. After the removal of the platform, the latency time J) decreased, and K) frequency, L) zone time increased significantly by at least double, respectively, during the sixth day, indicating that the memory ability was recovered by at least 60% after treatment. (*n* = 6). Statistical significance: *P < 0.05, **P < 0.01, ***P < 0.001, and ****P < 0.0001.

Based on the great improvement of inflammation and neuronal differentiation in TBI rats after treatment with NACHP hydrogel, the pathological lesions, synaptic reconstruction, recovery of behavioral and cognitive functions, and spatial learning that is highly dependent on hippocampal function were finally explored. 28 d after injury, the circular defect caused by the TBI group was the largest, and the degree of defect in the NACHP group was significantly reduced. H&E staining showed (**Figure** **7**A) that obvious cavitation appeared in the gray matter of the right upper brain of the TBI group, and the order of cavitation from large to small was: TBI group>NSC group>NACH group>NACHP group>Sham group, indicating that NACHP has a good effect on tissue structure repair. Moreover, following treatment with NACHP, no significant morphological differences were observed in brain tissue or other major organ tissues, including the heart, liver, lungs, spleen, and kidneys (Figure S25, Supporting Information). This finding indicates that NACHP exhibits excellent biocompatibility and poses no risk of tumorigenesis.

Immunofluorescence staining for presynaptic SYN and postsynaptic PSD95 (Figure 7B; Figure S23, Supporting Information) showed increased positive rates in the NACHP group compared to the NSC and NACH groups. The NACHP group exhibited the highest increase in SYN and PSD95, indicating its superior ability to promote synaptic reconstruction. At 4 weeks after transplantation, whole‐cell patch‐clamp recordings were performed on grafted GFP⁺ cells (*n* = 10 per group) (Figure S24A–C, Supporting Information). In the NACHP group, evoked action potentials were observed in 3 out of 10 GFP⁺ cells (Figure 7C; Figure S24D, Supporting Information), and excitatory postsynaptic currents (EPSCs) were detected in 1 cell, demonstrating functional synaptic integration with host neural circuits. In contrast, no action potentials or synaptic currents were recorded from any GFP⁺ cells in the NSC group. The sustained firing activity in responsive cells illustrates the successful integration of transplanted NSCs following NACHP treatment. Rats with moderate‐to‐severe TBI (mNSS score > 8 at 24 h postinjury) were selected for subsequent experiments to ensure injury consistency. At 28 d post‐treatment, all therapeutic groups (NSC, NACH, NACHP) exhibited progressive recovery trends in sensorimotor functions, with the NACHP cohort demonstrating the most pronounced improvement. (Figure 7D).

28‐d post‐treatment exploration of rats (Figure 7E), highlighting a marked reduction in TBI group activity compared to the Sham group in the open field test. This suggests heightened anxiety or impaired mobility post‐injury. The NSC, NACH, and NACHP treatments evinced a progressive recovery, with NACHP yielding the most robust improvement. In open field experiments, the activity distance in the central area serves as a key indicator to assess exploratory behavior, anxiety levels, and mobility. As shown in Figure 7F, prior to modeling (day‐1), there was no significant difference in the central area distance among all groups, ensuring the balance and comparability of the experiment. At day 7, a significant decrease in activity across all groups indicated a successful establishment of the TBI model. At day 28, although the TBI group showed signs of natural recovery, their activity distance was still significantly lower than that of the other four groups. The recovery in the NSC group was further enhanced, and the NACH group continued to show signs of improvement. The most remarkable result was seen in the NACHP group, which displayed the most significant recovery effect at 28 d. These findings underscore the potential advantage of the combined treatment strategy in promoting the improvement of behavioral indicators following brain injury.

Subsequently, we determined the degree of macro neurocognition through the Morris water maze task and evaluated the recovery of learning and memory ability after TBI in rats (Figure 7G). During the five‐day training, the time to seek the platform (escape latency) and the length of the path gradually decreased. These were the main parameters that reflected the spatial learning ability of TBI rats. During the five‐day training, the time to reach the platform of the NACHP group decreased more than that recorded in the NSC and NACH groups (Figure 7I). This indicated that the device can effectively enhance the learning ability of TBI rats. On day 6, a probe test was performed without the platform to assess the memory consolidation and memory retrieval ability of rats. The results of the probe test showed that the rats in the NACHP group could remember the position of the platform, as indicated by the swimming path (Figure 7H). In contrast, the rats in the NSC and NACH groups showed more chaotic swimming paths during the test. Compared to the rats in NSC and NACH groups, the latency time (Figure 7J) of the NACHP group was significantly decreased, and the frequency (Figure 7K) and zone time in the target quadrant (Figure 7L) were significantly increased by at least double, respectively. These findings indicated that the NACHP can make memory functional recovery by at least 60% following TBI.

Our ACHP transducer exhibits a unique dual functionality: on one hand, it remodels the immune microenvironment through a PDA coating, and on the other hand, it provides pro‐regenerative electrical signals via ultrasound‐driven piezoelectric effects, creating a favorable niche for the survival and neurogenesis of NSCs.^[^ ^36^ ^]^ In contrast to traditional wired deep brain stimulation (DBS) systems, this wireless and self‐powered platform eliminates the need for implanted batteries or leads, significantly enhancing the feasibility and safety of clinical translation.^[^ ^35^ ^]^ Furthermore, the PDA coating not only enhances piezoelectric output performance but also actively scavenges ROS and suppresses inflammatory responses, achieving genuine synergy among anti‐inflammatory action, electrical stimulation, and neural regeneration‐rather than mere functional component addition.

This multimodal therapeutic strategy demonstrates significantly superior therapeutic outcomes by synergistically addressing key bottlenecks in current TBI treatment. Its immunomodulatory component effectively alleviates the inflammatory microenvironment that is hostile to regeneration,^[^ ^45^ ^]^ while the wireless localized electrical stimulation directly promotes neurogenesis and synaptic integration.^[^ ^49^ ^]^ The biodegradable nature and self‐powering capability of the system further support its translational potential in the treatment of traumatic brain injury, and provide a novel technological pathway for treating other neurological disorders such as cerebral infarction and Alzheimer's disease.^[^ ^19^ ^]^

### 2.8 Investigation of Underlying Mechanisms

To further investigate the intrinsic mechanisms underlying the aforementioned behavioral improvements, we conducted an in‐depth study of the peri‐lesion area through transcriptome sequencing and molecular biology experiments. The results demonstrate that NACHP synergistically promotes neurological functional recovery through the “immune‐neuro” dual‐modulation (Figure S26, Supporting Information).

In the early injury phase (7 d), NACHP significantly modulated the neuroimmune microenvironment. It has been reported that early neuroinflammatory responses and impaired neural plasticity following TBI are key factors contributing to anxiety‐like behaviors and cognitive dysfunction. alleviating neuroinflammation can directly ameliorate anxiety‐like behaviors and promote the recovery of cognitive function.^[^ ^2^, ^34^, ^45^ ^]^ The volcano plot revealed a distinct set of DEGs between groups at this stage (Figure S26A, Supporting Information). The heatmap visualization specifically illustrated upregulation of anti‐inflammatory and neural development‐related genes (Figure S26B, Supporting Information). KEGG analysis further demonstrated that these DEGs were primarily enriched in immune‐related pathways including the NOD‐like receptor signaling and NF‐κB signaling pathways, as well as neural‐related pathways such as the PI3K‐Akt signaling pathway and Neuroactive ligand‐receptor interaction pathway (Figure S26C, Supporting Information). qRT‐PCR validation showed downregulation of the pro‐inflammatory gene C4B and upregulation of immunomodulatory genes IL2RG and CD55 (Figure S26D, Supporting Information), indicating that NACHP effectively suppresses excessive inflammatory responses at the early stage. Concurrently, neural development‐related genes FN1 and ANGPT2, as well as the neuropeptide TRH, were significantly upregulated (Figure S26D, Supporting Information), suggesting that early immunomodulation creates a favorable environment for neural repair.

During the recovery phase (28 d), the mechanistic focus shifted toward neural regeneration and circuit remodeling. The volcano plot displayed DEGs at this stage (Figure S26E, Supporting Information). The heatmap analysis specifically highlighted expression patterns of genes associated with brain functional recovery, showing downregulation of inflammatory genes, upregulation of anti‐inflammatory genes, and enhanced expression of neural function‐related genes (Figure S26F, Supporting Information). KEGG analysis indicated sustained activation of the PI3K‐Akt signaling pathway and the neuroactive ligand‐receptor interaction pathway (Figure S26G, Supporting Information). Gene expression validation confirmed that IL2RG and TRH remained significantly upregulated, while the nuclear receptor NR4A1 was markedly downregulated (Figure S26H, Supporting Information). Notably, IL2RG, a key factor in the PI3K‐Akt pathway, was differentially expressed at both 7 and 28 d (Figure S26A,E, Supporting Information), potentially facilitating repair through its dual role in modulating immune cell function and neurogenesis.^[^ ^50^ ^]^ The neuropeptide TRH, which was also highly expressed at both time points (Figure S26D,H, Supporting Information), is presumed to enhance cognitive function by regulating neuronal excitability and synaptic function.^[^ ^51^ ^]^ The downregulation of NR4A1 may indicate its feedback regulatory role as a transcriptional repressor during the neural regeneration stage.

In summary, NACHP combination therapy operates through an “immune‐neuro” dual‐modulation mechanism. In the acute phase, it focuses on reprogramming the immune microenvironment to mitigate neuroinflammation; during the recovery phase, it promotes neurogenesis and synaptic remodeling by activating neurotrophic pathways and key genes via electrical stimulation, thereby reducing anxiety‐like behaviors, enhancing spatial learning and memory, and ultimately leading to significant recovery of behavioral and cognitive functions.

## 3 Conclusion

To summarize, we innovatively designed and fabricated immuno‐piezoelectric transducer. The transducer's micro‐nano fiber directional structure significantly enhanced the proportion of piezoelectric β‐phase and improved charge separation efficiency. Functional modification with PDA further accelerated charge separation and amplified the piezoelectric transduction effect. Through ultrasound‐induced wireless electronic stimulation, the transducers promoted the differentiation of NSCs into glutamatergic and GABAergic neurons with increased neurite complexity and lengths, indicating advanced neural maturation. Notably, this approach also inhibited differentiation of NSCs into glial cells. In the context of TBI treatment, the sustained action of PDA in the transducer promoted the transition of microglia towards an anti‐inflammatory M2 phenotype, increasing their numbers, vitality, and the secretion of anti‐inflammatory factors. This positively regulated the inflammatory microenvironment, creating conditions conducive to the survival of NSCs in vivo. More importantly, the transducer, in synergy with NSCs, reduced reactive gliosis and promoted the differentiation of transplanted NSCs into mature, Neun‐positive neurons. This was accompanied by elevated expression of pre‐ and postsynaptic membrane proteins, enabling integration and functional connectivity in the damaged area. These effects translated into a significant reduction in the injury area and notable improvements in learning, cognitive, and motor functions postbrain injury. Or our findings contribute to the development of minimally invasive treatment and can be translated into other relevant clinical neuro‐related disorders such as cerebral infarction, Alzheimer's disease, and depression, and advance the clinical application of those techniques.

## 4 Experimental Section

### Chemicals and Reagents—Synthesis of Wireless Immunoregulatory Piezoelectric Transducers

Lithium hydroxide (LiOH) and urea provided by Aladdin. Epichlorohydrin (ECH) is obtained by MREDA. Dopamine and concentrated sulfuric acid (H_2_SO_4_) were purchased from Sigma‐Aldrich. Tris‐Hcl buffer (pH = 8.8) was provided by Macklin. The filter paper is completely dried at 60 °C for use without further purification. Deionized water was used throughout the study.

### rNSCs Culture

DMEM/F12, Neurobasal, fetal bovine Serum (FBS), B27 and N2 supplements, l‐glutamine, trypsin, phosphate buffered saline (PBS, pH 7.2), and penicillin–streptomycin purchased from Gibco (Thermo Fisher Scientific, USA). Epidermal growth factor (EGF) and basic fibroblast growth factor (bFGF) were provided by Peprotech Inc (USA). Trizol reagent was purchased from Life Technologies (Thermo Fisher Scientific, USA). Neun, MAP2, PSD95, SYN, GFAP, Iba1, Ki67, Nestin, PAX6, SOX2, SOX1 primary antibodies and secondary antibodies were obtained from Abcam, VGLUT1 and VGAT primary antibodies were obtained from Synaptic Systems (SYSY, Germany). CD16/32 was purchased from BD Biosciences (USA). CD206 was purchased from Cell Signaling Technology (CST, USA). 4′,6‐Diamidino‐2‐phenylindole (DAPI) was supplied by Sigma‐Aldrich Chemical Co. (St. Louis, MO, USA). And Phalloidin was purchased from Solarbio (China). Trypsin inhibitors were supplied by OKA (China). Live/dead stain kits were provided by Invitrogen (Thermo Fisher Scientific, USA). The lentiviral vector pcSLenti‐EF1‐EGFP‐F2A‐Puro‐CMV‐MCS‐WPRE was constructed by OBiO Technology (Shanghai) Corp., Ltd. (China). Palomid 529 and BEZ235 were purchased from Selleck Chemicals (USA).

### Preparation of Immuno‐Piezoelectric Transducers

Anisotropic cellulose hydrogels are based on previously reported methods with some modifications.^[^ ^52^, ^53^ ^]^ A transparent cellulose solution (2 wt%) was prepared by dissolving the filter paper sheet in a 4.5 wt% LiOH/ 15 wt% urea solution and precooling to −20 °C. A certain amount of ECH was dropped into the above transparent cellulose solution and stirred evenly to conduct a preliminary chemical crosslinking reaction. The transparent viscous cellulose solution containing different ECH/AGU molar ratios (1, 1.25, 1.5, 1.75, 2, 2.25, 2.5) was centrifuged to remove bubbles, then poured into a 6 cm plastic petri dish and reacted in a refrigerator at 4 °C for 30 h. The loose chemically crosslinked cellulose hydrogels (LCC) were obtained, which were defined as LCC‐1, LCC‐2, LCC‐3, LCC‐4, LCC‐5, LCC‐6, and LCC‐7, respectively. The LCC is then prestretched to specific length ratios (0%, 40%, 80%, and 120%) and immersed in a 1–5 wt % H_2_SO_4_ solution within 1 min to terminate the chemical crosslinking reaction. At the same time, the nanofibers form physical crosslinking networks and obtain hydrogels with highly oriented structures through hydrogen bonding. Anisotropic cellulose hydrogels (ACH) with permanent shape were obtained after thorough washing with secondary water, and were defined as CH, ACH‐2, ACH‐3, and ACH, respectively.

Modified ACH (ACHP) with different contents of polydopamine (PDA): A layer of PDA was modified on the surface of ACH by in situ polymerization. The specific steps are as follows: the ACH is immersed in Tris‐Hcl buffer with pH of 8.8, and then dopamine is added at the concentration of 1, 2, and 4 mg mL^−1^, respectively. Rinse with water repeatedly and terminate the reaction after 2 h, coded as ACHP1, ACHP2, and ACHP4.

### Isolation and Culture of NSCs

Healthy Spregue Dawley (SD) pregnant mouse, at embryo day 16, were purchased from Beijing Sibeifu Biotechnology Co., LTD. The dural and associated blood vessels on the surface of the embryo forebrain were gently separated. The extracted forebrain tissue was then minced using tweezers in 4 °C sterile PBS. A single‐cell suspension was digested with 0.05% trypsin at 37 °C, followed by the addition of trypsin inhibitors at a 1:1 volume ratio to stop the digestion process. The cell suspension was then passed through a cell strainer (100 µm above and 200 µm below) to remove impurities and undigested tissue clumps. After counting and centrifugation, the cells were seed at a density of 2 × 10⁵ cells mL^−1^ and cultured for five days. The NSCs proliferation medium consisted of DMEM/F12 supplemented with 2% B27, 1% N2, 20 ng mL^−1^ bFGF, and 20 ng mL^−1^ EGF. For experimental reliability, second‐ to fourth‐generation NSCs were used.

### Wireless Electronic Stimulation of NSCs

After sterilization of the ACH and ACHP, an 8 µL cell suspension containing 5×10⁵ cells was seeded onto materials in the 24‐well plate. Following a 20‐min incubation, 1 mL of NSCs proliferation medium was added. After 24 h, once the NSCs had fully attached, the medium was replaced with differentiation medium. The NSCs differentiation medium consisted of Neurobasal medium supplemented with 1% l‐glutamine, 2% B27, 1% penicillin/streptomycin, and 1% fetal bovine serum. The culture plates were then sealed and subjected to ultrasound stimulation using 400 W of ultrasound energy at a frequency of 20 kHz (8 min/time, twice daily). NSC was used as a negative control for this experiment.

### Biocompatibility of NSCs

To visually monitor the survival status of NSCs on ACH and ACHP piezoelectric hydrogels under different wireless ultrasound stimulation, live/dead staining was performed according to manufacturer's instructions. After 2 d of different wireless ultrasound stimulation, 200 µL sterile PBS containing 2 × 10^−6^ m calxanthin and 4 × 10^−6^ m propyl iodide was used for incubation at 37 °C for 10 min. After three times of PBS washing, NSCs survival was observed using a confocal laser microscope.

### RNA Isolation and Reverse Transcription Quantitative Real‐Time PCR (RT‐qPCR)

Total RNA was isolated by Trizol reagent according to the manufacturer's instructions. cDNA was synthesized using PrimeScript RT Master Mix (TAKARA, RR036A) according to manufacturer's protocol. Each qPCR reaction contained 1× LightCycler 480 SYBR Green I Master (Roche, #4 887 352 001), 1 × 10^−3^ m primers (Forward and Reverse) and 0.2 µL cDNA. RT‐qPCR Primer pairs were designed to span an exon–exon junction and/or at least one intron. Target sequences were amplified using the LightCycler 2.0 Real‐Time PCR system (Roche Life Science) with the following amplification protocol: 5 min at 95 C followed by 45 cycles of 10 s at 95 C, 20 s at 60 C, and 20 s at 72 C. Data analysis was performed using LinRegPCR program.50 Relative expression values were obtained by normalizing N0 values to the Geomean of experimentally assessed reference genes GAPDH. Primers used in this study are listed in Table S1 in the Supporting Information.

### Cellular ROS Scavenging Ability of ACHP

5×10^5^ NSCs cells were seeded onto each hydrogel sample (*Φ* = 15 mm). After 7 d of differentiation, the cells were exposed to 200 × 10^−6^ m H_2_O_2_. Following 3 d of ultrasound treatment, the cells were rinsed three times with serum‐free medium. Subsequently, a 10 × 10^−6^ m solution of DCFH‐DA and DHE probes (Beyotime, Shanghai, China) was introduced into the medium and incubated with the cells for 30 min in darkness. Finally, the cells underwent three additional washes with serum‐free medium prior to analysis. Cellular ROS levels were assessed using a fluorescence microscope and quantified with a microplate reader.

### Anti‐inflammatory Ability of ACHP

3×10^5^ BV2 cells were seeded onto hydrogels (*Φ* = 15 mm) and cultured for 12 h before the medium was replaced with fresh medium containing 2 mg mL^−1^ LPS. After 2 d of ultrasound treatment, BV2 cell transformation was observed using CLSM (Carl Zeiss LSM900). mRNA expression levels of pro‐inflammatory cytokines, including IL‐13 and IL‐6, were quantified in BV2 cells via qPCR using primers sourced from Sangon Biotech (Shanghai, China), Primers are listed in Table S1 in the Supporting Information.

### RNA Sequencing and Analysis of Differentially Expressed Gene

RNA sequencing was completed by Bestopcell Technology Company (Beijing). The experimental process mainly includes sample detection, library construction and quality inspection, and computer sequencing. Briefly, NSCs were cultured in the differentiation medium for 7 d, the total RNA was extracted by Trizol reagent according to the manufacturer's instructions. Subsequently, the purity and concentration of RNA was detected by NanoDrop 2000 spectrophotometer, and the integrity of RNA was accurately detected by Agient2100/LabChip GX. After the sample test was qualified, the library was constructed. Qubit 3.0 fluorescence quantifier was used for preliminary quantification, and the concentration should be above 1 ng µL^−1^. Then, Qsep400 high‐throughput analysis system was used to detect the inserted fragments of the library. When the effective concentration (>2 × 10^−9^ m) of the library was qualified by qPCR, Illumina NovaSeq6000 sequencing platform was used for PE150 mode sequencing.

### Immunohistochemistry

Tissues were fixed overnight in 4% paraformaldehyde solution and transferred to 30% sucrose before being snap‐frozen in O.C.T (Fisher Scientific, Pittsburgh, PA). Cells in culture were directly fixed in 4% paraformaldehyde for 30 min, followed with 60 min of permeabilization and blocking in PBS supplemented with 0.2% Triton X‐100 and 5% horse serum. For immunofluorescence, cells or tissue sections were stained with primary antibodies at 4 °C overnight and secondary antibodies at room temperature for 1 h. The information for primary antibodies and secondary antibodies were provided in Table S3 in the Supporting Information. Nuclei were counterstained by DAPI.

### In Vivo Study of NACHP in TBI Treatment

A traumatic brain injury (TBI) model using fluid impact was established in 8‐ to 10‐week‐old male Sprague‐Dawley (SD) rats weighing 180–200 g. Each rat was anesthetized by intraperitoneal injection. After the anesthesia was successful, the rats were fixed on the stereotactic instrument, the scalp was cut on the right side of the median line of the head, and the skull was drilled with a dental drill at the side of the middle point of the left fontanel line. Mosquito type vascular clamp was used to enlarge the bone window and form a bone window with a diameter of 5 mm. The dural membrane was maintained intact. A free fall striking device was adopted, and the impact pin fell from a height of 25 cm with a weight of 60 g acted on the brain tissue, and contusion of the left parietal lobe was caused when the iron block fell less than 1 mm. The percussion rod was removed immediately after the blow, and the sterile cotton ball was dipped into the external blood of the dry dural membrane to reveal the dural membrane. After the modeling was successful, the rats were fed in a clean, quiet, 20 °C, avoiding strong light. Subsequently, the rats with brain injury were randomly divided into five groups (*n* = 6 per group): Sham group, TBI group, NSC group, NACH group, NACHP group. NSCs were transduced with pcSLenti‐EF1‐EGFP‐F2A‐Puro‐CMV‐MCS‐WPRE lentiviral vector (Obio Technology, Shanghai). GFP‐NSCs (4×10^5^ per rat) were first injected into the site of brain injury. After that, ACHP were adhered to the injury site of the rats. Two days post‐injection, and the rats were subjected to wireless electrical signals induced by ultrasound (2 W cm^−2^, 1 MHz) for 7 d. Finally, the rats were euthanized, and the TBI repair was evaluated by H&E staining, Nissl staining, immunofluorescence staining and behavioral tests according to the manufacturer's instructions. Detailed sample sizes per experimental group, including time‐course analyses, functional assessments, and mortality data, are summarized in Table S2 in the Supporting Information.

### Open Field Test

The open field test measures rat exploratory behavior in novel environments, performed before TBI and at 7 and 28 d post‐TBI. Rats acclimated to the test room a day prior to reduce environmental effects. The 60 cm central area of the chamber is critical, with the rest as periphery. A quiet, well‐lit setting ensures accurate results. Rats' 10‐min trajectories in the center are recorded, and the chamber is cleaned post‐test. Labmaze software analyzes central zone movement to assess motor skills and exploration interest.

### Morris Water Maze Experiments

Morris water maze was used to assess learning and cognitive function. The water maze is divided into four quadrants, with the platform located at the center of Quadrant III. The rats were placed in the pool in the sequence I→II→IV quadrants. They were trained to find the hidden platform as an exit and remained on it for 10 s. The training lasted for five consecutive days.

Following the training phase, a probe test was conducted with the platform removed. During this test, the rats were allowed to swim freely for 60 s, while measurements were taken for the distance to the former platform location, the duration spent in the platform's area, and the frequency of crossing the platform location.

### Statistical Analysis

All data are expressed as mean ± standard deviation (SD). Multivariate statistical analysis was performed by one‐way ANOVA and GraphPad Prism software, and *P* values < 0.05 were considered statistically significant.

## Conflict of Interest

The authors declare no conflict of interest.

## Supporting information



Supporting Information

## Data Availability

The data that support the findings of this study are available from the corresponding author upon reasonable request.
