# Supplementary material for: Neuroimmune Microenvironment Reprogramming via Immuno‐piezoelectric Transducers for Synergistic Stem Cell Therapy in Traumatic Brain Injury
Source: Adv Mater. 2025 Oct 1;38(2):e12810. doi: 10.1002/adma.202512810 (PMC12783878; doi:10.1002/adma.202512810)
Supplement: Supplementary file 1 — Supporting Information [file ADMA-38-e12810-s001.doc]

**Supporting Information**

**Neuroimmune Microenvironment Reprogramming via Immuno-piezoelectric Transducers for Synergistic Stem Cell Therapy in Traumatic Brain Injury**

**Linlin Liang^a,†^, Xin Li^a,†^, Kai Hu**^d^**, Pingqiang Cai^c^, Jianwu Wang^c^, Jing Yu^c^, Shasha Wang^a^, Yuwei Zhao**^a^**, Changgeng Xu**^a^**, Siwei Li**^a^**, Hong Liu^b,*^, Changyong Wang^a,*^ and Jin Zhou^a,*^**

^a^*Beijing Institute of Basic Medical Sciences, 27 Taiping Rd, Beijing 100850, PR China*

^b^*State Key Laboratory of Crystal Materials, Shandong University, 27 Shandanan Road, Jinan, Shandong, 250100, PR China. Institute for Advanced Interdisciplinary Research University of Jinan, Jinan, Shandong 250022, PR China*

^c^*Digital Molecular Analytics and Science (IDMxS), Nanyang Technological University, 59 Nanyang Drive, Singapore 636921, Singapore.*

*^d^The First School of Clinical Medicine, Southern Medical University,Guangzhou,Guangdong, 510515, PR China*

**^†^***These authors contributed equally to this work.*

E-mail addresses: zhoujin@bmi.ac.cn (J. Zhou), wangchy@bmi.ac.cn (C. Wang), [hongliu@sdu.edu.cn](mailto:hongliu@sdu.edu.cn) (H. Liu)

**Experimental Section**

**S1. Chemical crosslinking molecular formula**

Under alkaline conditions, the crosslinking between ECH and cellulose is primarily achieved through an epoxide ring-opening-etherification reaction. The main reaction formula is as follows:

Cellulose-OH + OH⁻ → Cellulose-O⁻ + H_2_O

Cellulose-O⁻ + H_2_C-CH-CH_2_Cl → Cellulose-O-CH_2_-CH(OH)-CH_2_Cl

Cellulose-O-CH_2_-CH(OH)-CH_2_Cl + ⁻O-Cellulose → Cellulose-O-CH_2_-CH(OH)-CH_2_-O-Cellulose + Cl⁻

**S2. *In vitro* degradation test**

Pre-weighed (W₀) ACHP hydrogels (n=3) were immersed in PBS (pH=7.4), collagenase solution (100 U/mL in PBS), and cellulase solution (25 U/mL in PBS), respectively, and incubated under shaking at 37 °C. At predetermined time points (1, 3, 5, 7 days), the samples were taken out, gently rinsed with deionized water, freeze-dried, and weighed again (Wₜ). The degradation ratio was calculated as follows:
Degradation ratio (%) = [(W₀ − Wₜ) / W₀] × 100%.

**S3. Subcutaneous and intracerebral biocompatibility test**

**Animal model and implantation**: Healthy SD rats were randomly divided into a Sham group (surgery only) and an ACHP implantation group (n=3/group at 3, 7, and 14 days). After anesthesia, a small subcutaneous pocket was created on the back for ACHP implantation in the subcutaneous group. For the intracerebral group, a small ACHP block was implanted into the cerebral cortex through a cranial drill hole.

**Tissue collection and analysis**: Skin or brain tissues from the implantation site were collected on days 3, 7, and 14 post-implantations. The tissues were fixed in 4% paraformaldehyde, embedded in paraffin (skin) or frozen (brain), sectioned, and subjected to hematoxylin and eosin (H&E) staining and immunohistochemical staining for CD86 (a marker for M1 macrophages/activated microglia). Inflammatory responses were observed under an optical microscope.

**S4. MRI Procedure**

To verify the consistency of the traumatic brain injury (TBI) model and evaluate the lesion volume, multimodal magnetic resonance imaging (MRI) scans were performed on day 3 post-modeling on rats (n=7) selected based on modified Neurological Severity Score (mNSS) > 8.

Animal preparation: Rats were anesthetized with isoflurane (4% for induction, 1.5–2% for maintenance) and secured in an animal-specific MRI holder. A heating pad was used to maintain body temperature at ~37 °C.

MRI scanning: MRI measurements were carried out on a 9.4 T small animal MRI system (uMR 9.4T, United Imaging Life Science Instrument, Wuhan, China) with a three-channel rat brain surface coil. The sequences included:

An anatomical scan was acquired using a fast spin-echo (FSE) 2D T2-weighted sequence with the following parameters: repetition time (TR) = 3000 ms, echo time (TE) = 45 ms, echo train length (ETL) = 13, field of view (FOV) = 35 × 35 mm^2^, matrix size = 232 × 232, slice thickness = 0.5 mm without gap, number of averages (NEX) = 2.

In-vivo diffusion-weighted images (DWI) were acquired using the echo-planar imaging (EPI) sequence with the following parameters: TR = 4000 ms, TE = 31 ms, b-values = 0, 800 and 1500 s/mm^2^, diffusion encoding directions = 3, FOV = 35 × 35 mm^2^, matrix size = 128 × 128, slice thickness = 0.5 mm, NEX = 4.

Apparent diffusion coefficient (ADC) map: Voxel-wise ADC maps were automatically generated based on DWI data acquired at multiple b values.

Image analysis: T2-weighted images were analyzed using ImageJ software (NIH). The hyperintense T2 regions (indicating lesions) on each slice were manually outlined to quantify the total lesion volume (mm^3^). The average volume ± standard deviation (SD) was calculated for all animals.

**S5. Patch-clamp procedure**

Used to record the electrophysiological activity of neurons differentiated from transplanted GFP-positive neural stem cells (NSCs) in vitro.

**Sample preparation**: After deep anesthesia, rats were perfused transcardially with ice-cold artificial cerebrospinal fluid (ACSF, composition below) saturated with 95% O_2_/5% CO_2_. The brain was quickly removed, and coronal sections of 300 μm thickness were cut in ice-cold oxygenated ACSF. Brain slices containing transplanted cells were recovered in ACSF at 32 °C for 1 hour, and then maintained at room temperature for at least 0.5 hour before recording.

**ACSF composition (in mM)**: 125 NaCl, 2.5 KCl, 1.25 NaH_2_PO_4_, 25 NaHCO_3_, 2 CaCl_2_, 1 MgCl_2_, 25 Glucose. Continuously aerated with 95% O_2_/5% CO_2_, pH 7.4.

**Intracellular pipette solution (in mM)**: 135 K-gluconate, 5 KCl, 0.5 EGTA, 10 HEPES, 2 Mg-ATP, 0.3 Na_2_-GTP, 10 Na_2_-phosphocreatine. pH adjusted to 7.4 with KOH, osmolarity 290-300 mOsm.

**Recording**: Brain slices were transferred to a recording chamber and continuously perfused with oxygenated ACSF at 32 °C (flow rate 2-3 mL/min). GFP-positive cells were identified under differential interference contrast (DIC) optics. Whole-cell recordings were made using borosilicate glass electrodes (resistance 4-6 MΩ). Signals were acquired with a Multiclamp 700B amplifier, low-pass filtered at 2 kHz, and sampled at 10 kHz using pCLAMP 10.7 software.

**Stimulation and data analysis**: Electrical stimulation was delivered via a bipolar tungsten electrode placed in the peri-lesion area to evoke excitatory postsynaptic currents (EPSCs). Spontaneous and evoked electrical activities, including action potentials and EPSC properties, were analyzed.


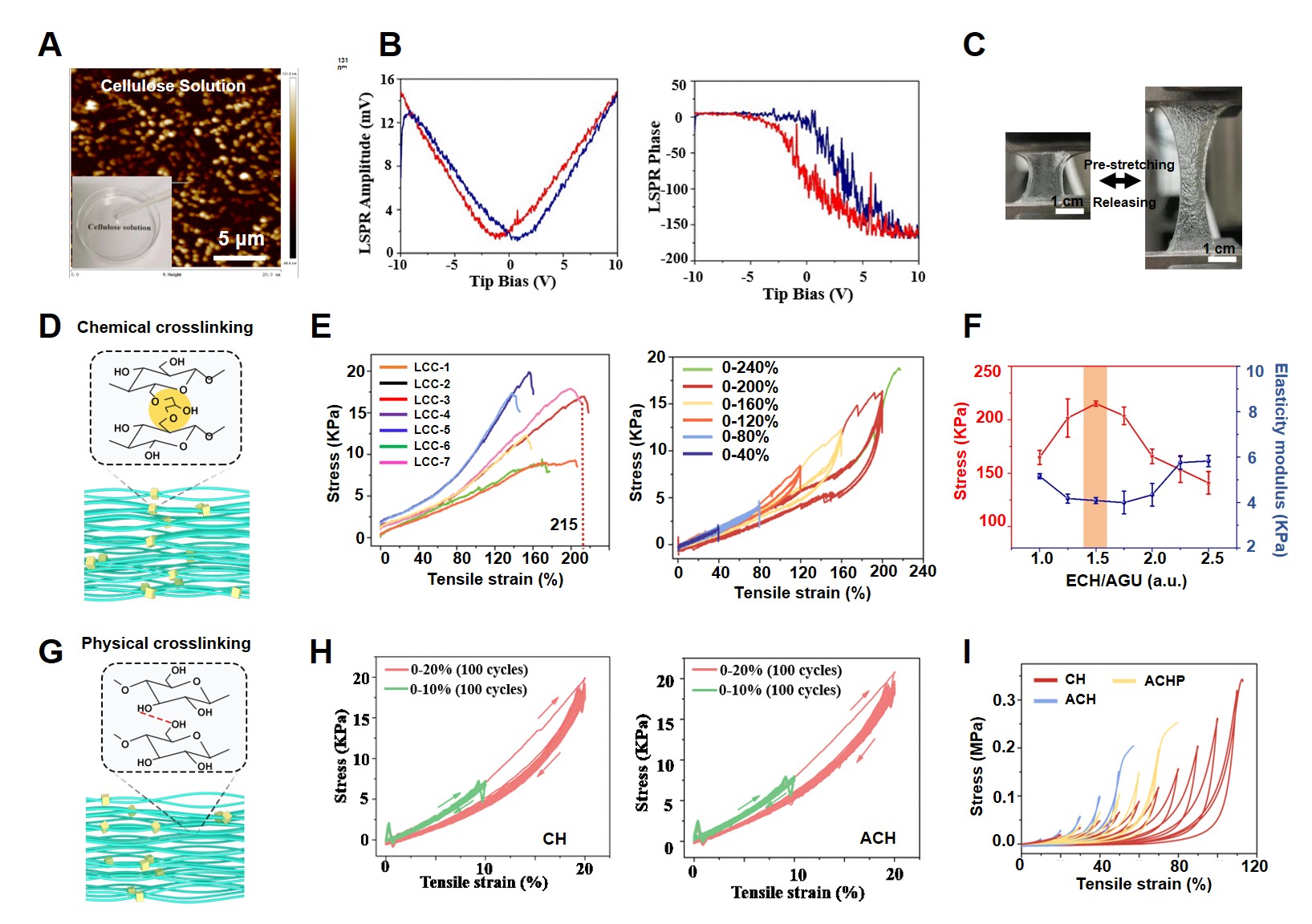


**Figure S1.** (A) AFM characterization of the cellulose solution, with a photograph of the alkali-dissolved cellulose solution insect. (B) The ferroelectric hysteresis loop (right) and butterfly-shaped amplitude loop (left) of the cellulose solution under a direct voltage range of -10 V to 10 V, confirming the piezoelectric properties of nanocellulose. (C) Photograph of the reversible stretching of ACH. (D) Schematic diagram of chemical crosslinking. (E) Stress-strain test corresponding to the maximum tensile strength of LCC-3 under chemically crosslinked cellulose hydrogels and loading-unloading cycles. (F) Characterization of the tensile and elastic modulus of different chemically crosslinked hydrogels. (G) Schematic diagram of physically crosslinked hydrogels. (H) Fatigue resistance testing of CH and ACH. (I) Stress-strain tests correspond to the maximum tensile strength of CH, ACH, and ACHP.


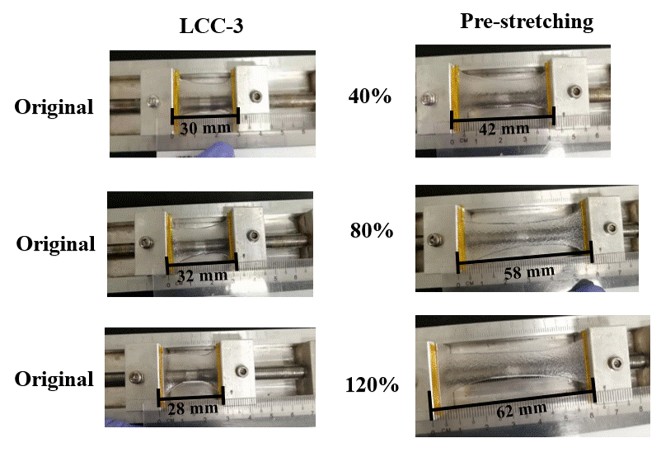


**Figure S2.** The process of stretching in different proportions.


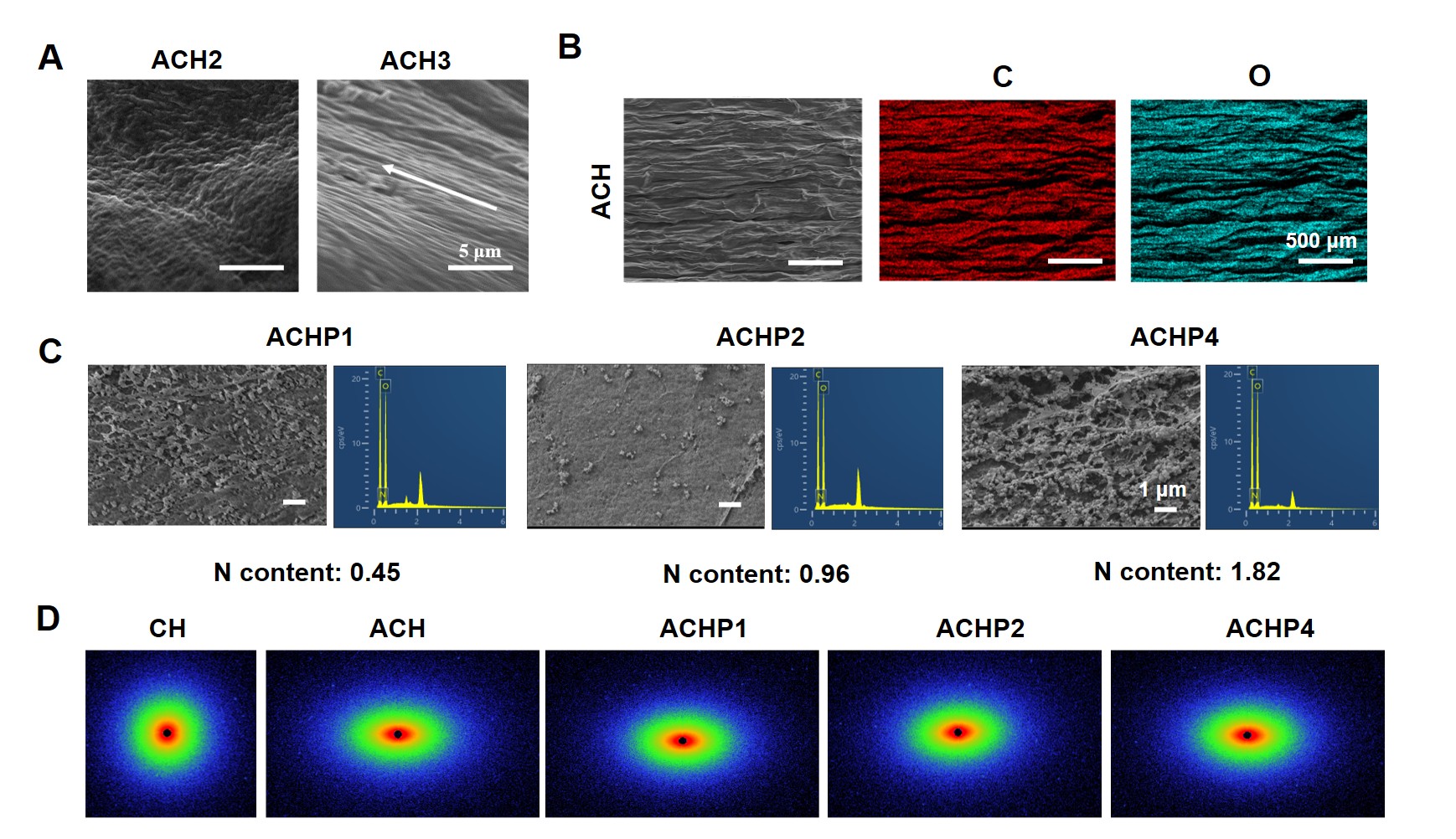


**Figure S3.** (A) SEM images of ACH2 and ACH3. (B) Elemental mapping of ACH, including oxygen (O) and carbon (C). (C) SEM and elemental analysis of ACHP1, ACHP2, and ACHP4. (D) Small-angle X-ray scattering characterization of various hydrogels.


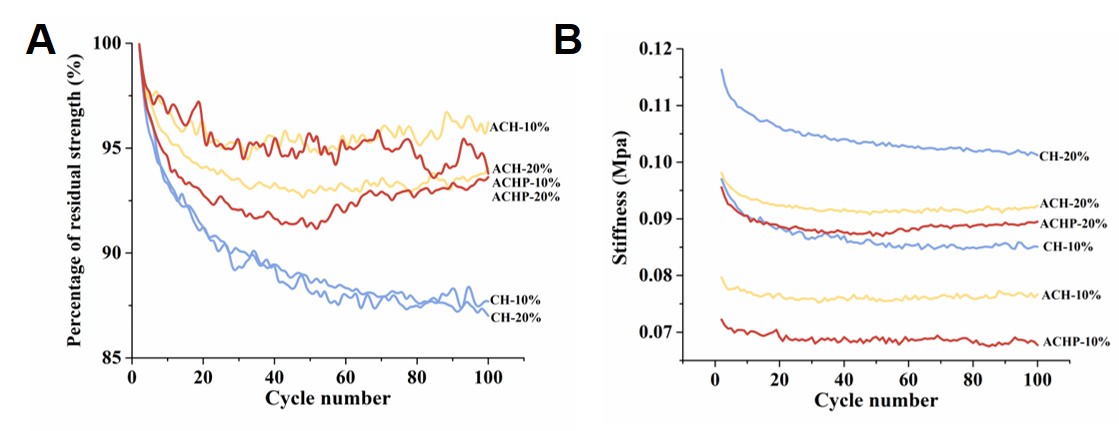


**Figure S4.** Quantitative analysis of cyclic anti-fatigue performance and basic mechanical properties of hydrogels. (A) Percentage of residual tensile strength relative to initial tensile strength for hydrogel samples in different groups after 100 cycles of tensile loading with 10% or 20% strain. (B) Retention rate of hydrogel stiffness relative to its initial stiffness during the corresponding cyclic tests.


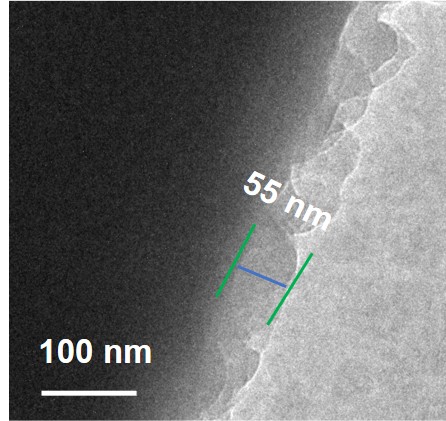


**Figure S5.** Transmission Electron Microscopy (TEM) images of the cross-section of ACHP


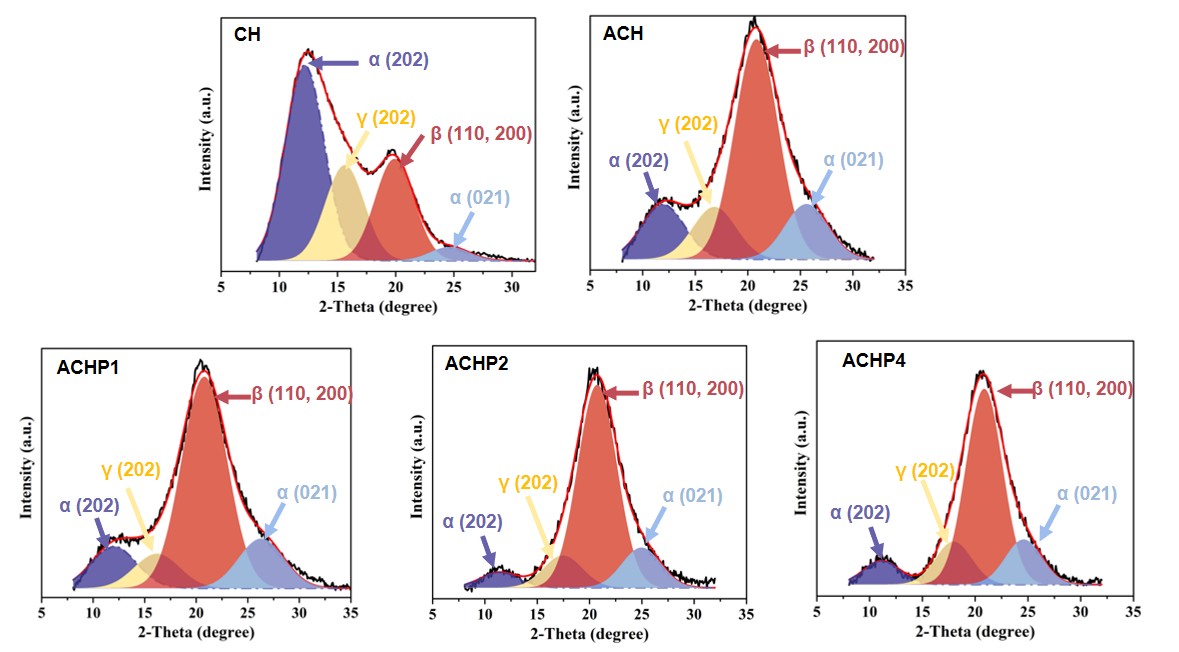


**Figure S6.** XRD peak fitting curves for various hydrogels, depicting the deconvolution of XRD spectra for CH, ACH, ACHP1, ACHP, and ACHP4 within the 2θ range of 8-35°. The peaks corresponding to the α, β, and γ phases are described, along with their respective lattice planes.


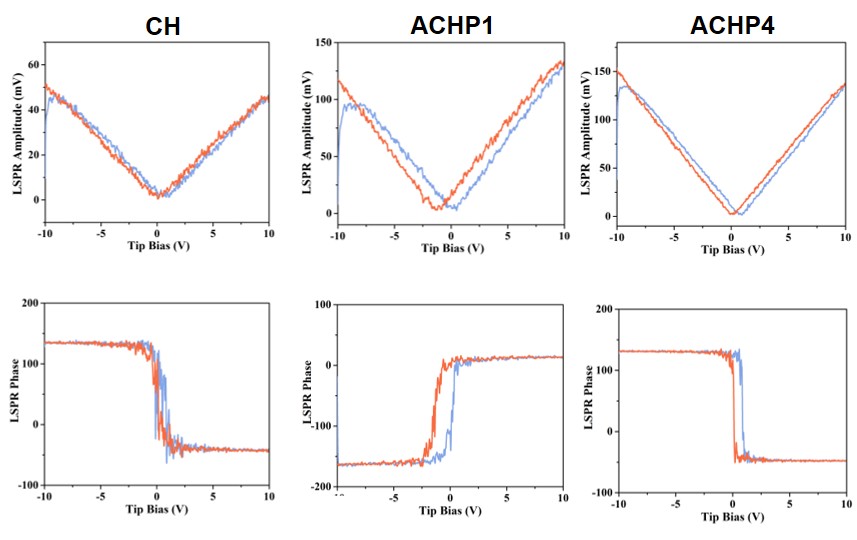


**Figure S7.** Ferroelectric hysteresis loops (bottom) and butterfly-shaped amplitude loops (top) of different hydrogels under a direct voltage range of -10 V to 10 V.


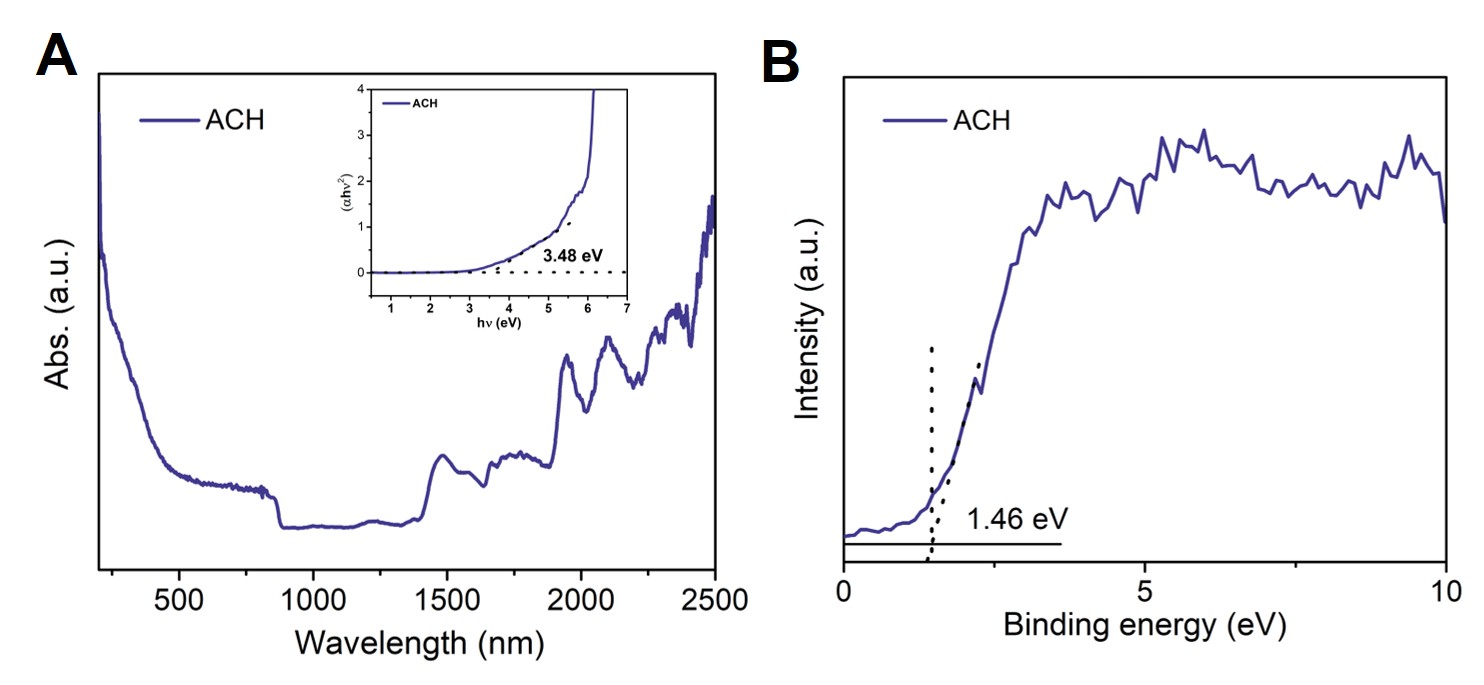


**Figure S8.** (A) Characterization of ACH by ultraviolet diffuse reflectance spectroscopy; (B) Valence Band X-ray Photoelectron Spectroscopy (VB-XPS) characterization of ACH.


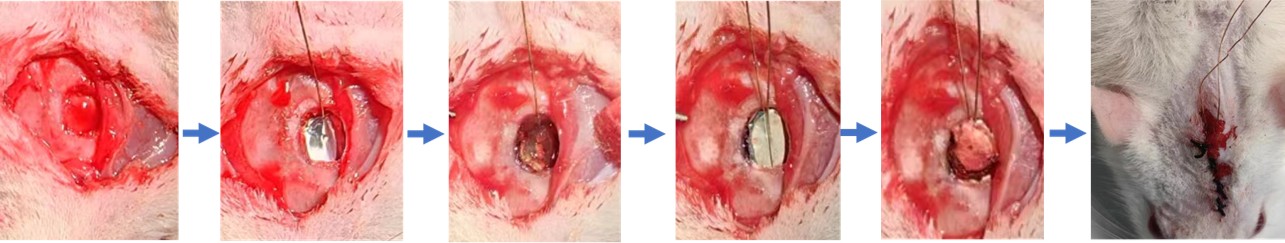


**Figure S9.** Piezoelectricity generated by ultrasound is implanted in vivo.


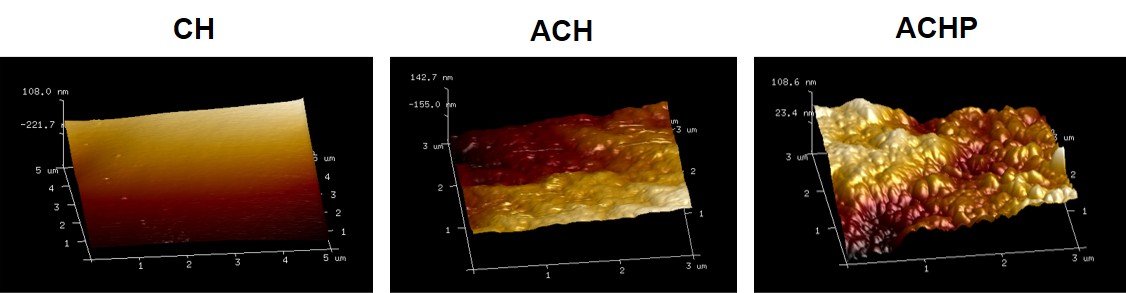


**Figure S10.** AFM Characterization of CH, ACH and ACHP.


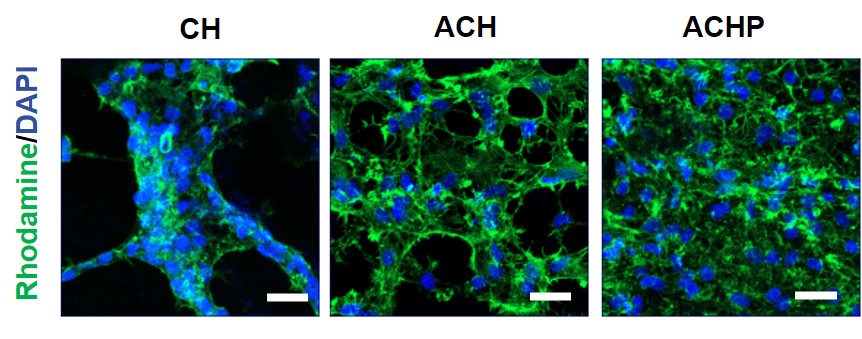


**Figure S11.** Phalloidin staining was performed on CH, ACH and ACHP for 2 days.


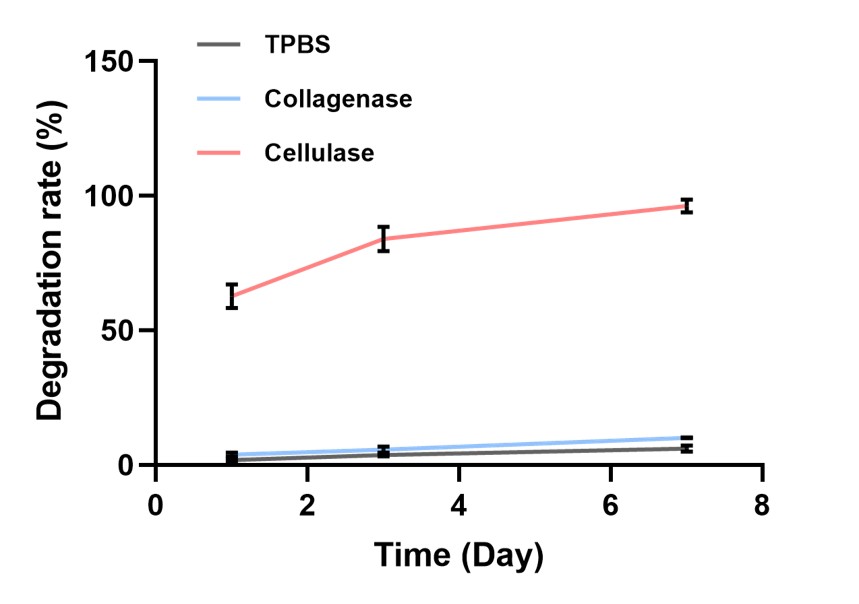


**Figure S12.** In vitro degradation curves of ACHP hydrogels under the action of TPBS, Collagenase, and Cellulase.


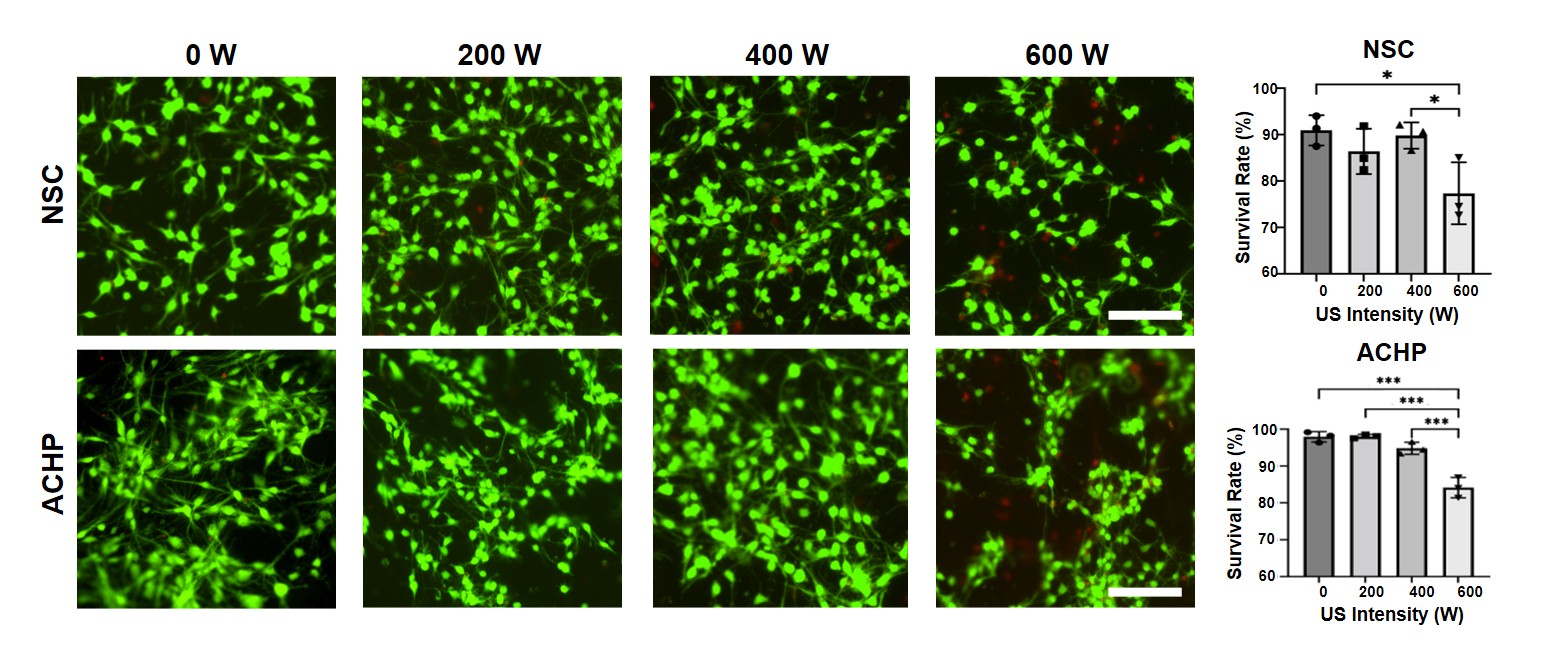


**Figure S13.** ACHP biocompatibility under varying ultrasonic intensities (green represents viable cells, red represents dead cells), scale bar: 50 µm.


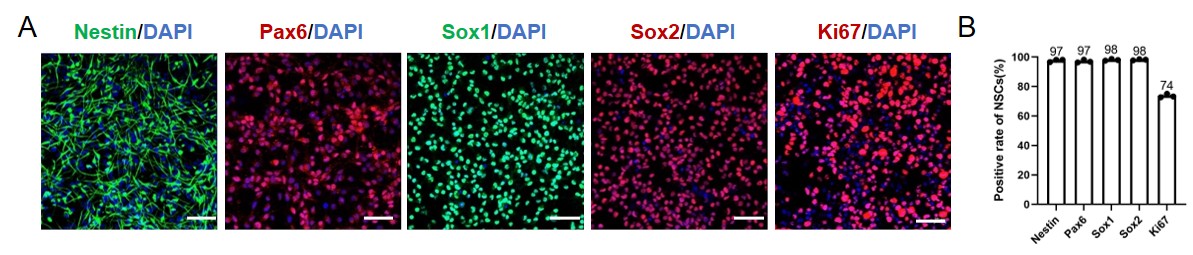


**Figure S14.** Characterization of NSC. (A) Immunofluorescence analysis of NSC marker proteins in NSC using specific antibodies directed to Nestin, Pax6, Sox1, Sox2 and proliferation marker Ki67. Scale bars, 50 μm. (B) Quantification of the positive percentage of NSC markers Nestin, Pax6, Sox1, Sox2 and proliferation marker Ki67 (n=3).


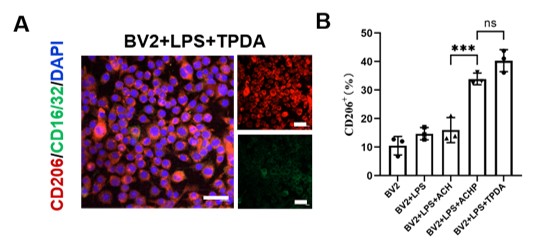


**Figure S15.** BV2 microglial polarization: Representative immunofluorescence images of BV2 microglial cells under lipopolysaccharide (LPS)-induced inflammatory conditions. CD206 (red) indicates M2 anti-inflammatory microglial polarization. CD16/32 (green) indicates M1 pro-inflammatory microglial polarization. DAPI (blue) marks the cell nuclei. n=3. Scale bar, 50 µm.


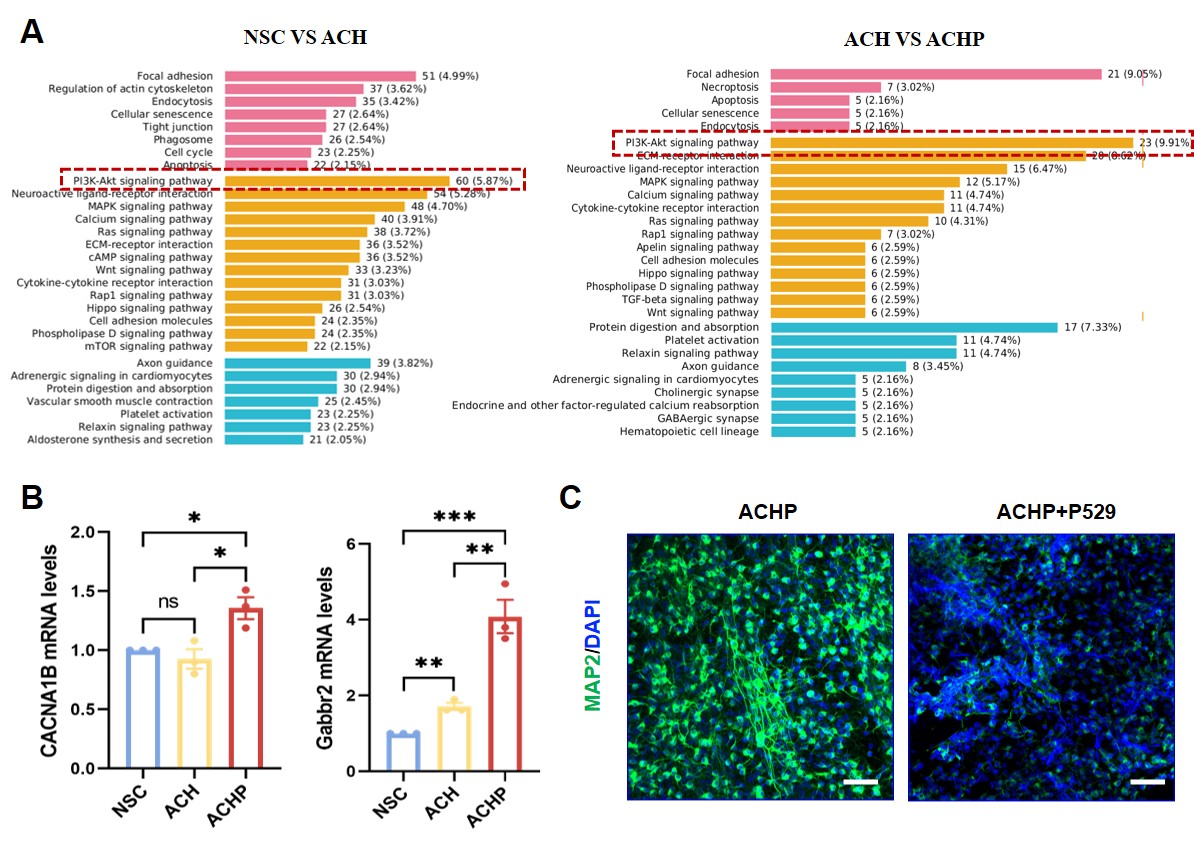


**Figure S16.** (A) The KEGG pathway classification of differentially expressed genes between NSCs cultured with wireless electrical stimulation on NSC, ACH and ACHP for 7 days.(B) Selected genes related to the neural differentiation that are upregulated in ACHP. Their respective fold changes measured by RNA-seq are as shown. **P* < 0.05, ***P* < 0.01, ****P* < 0.001, *****P* < 0.0001. (C) The immunofluorescence staining of mature neuron marker MAP2 (green) was performed after treatment with P529; scale bar: 50 µm. All data are expressed as the mean ±standard deviation (SD; n = 3).


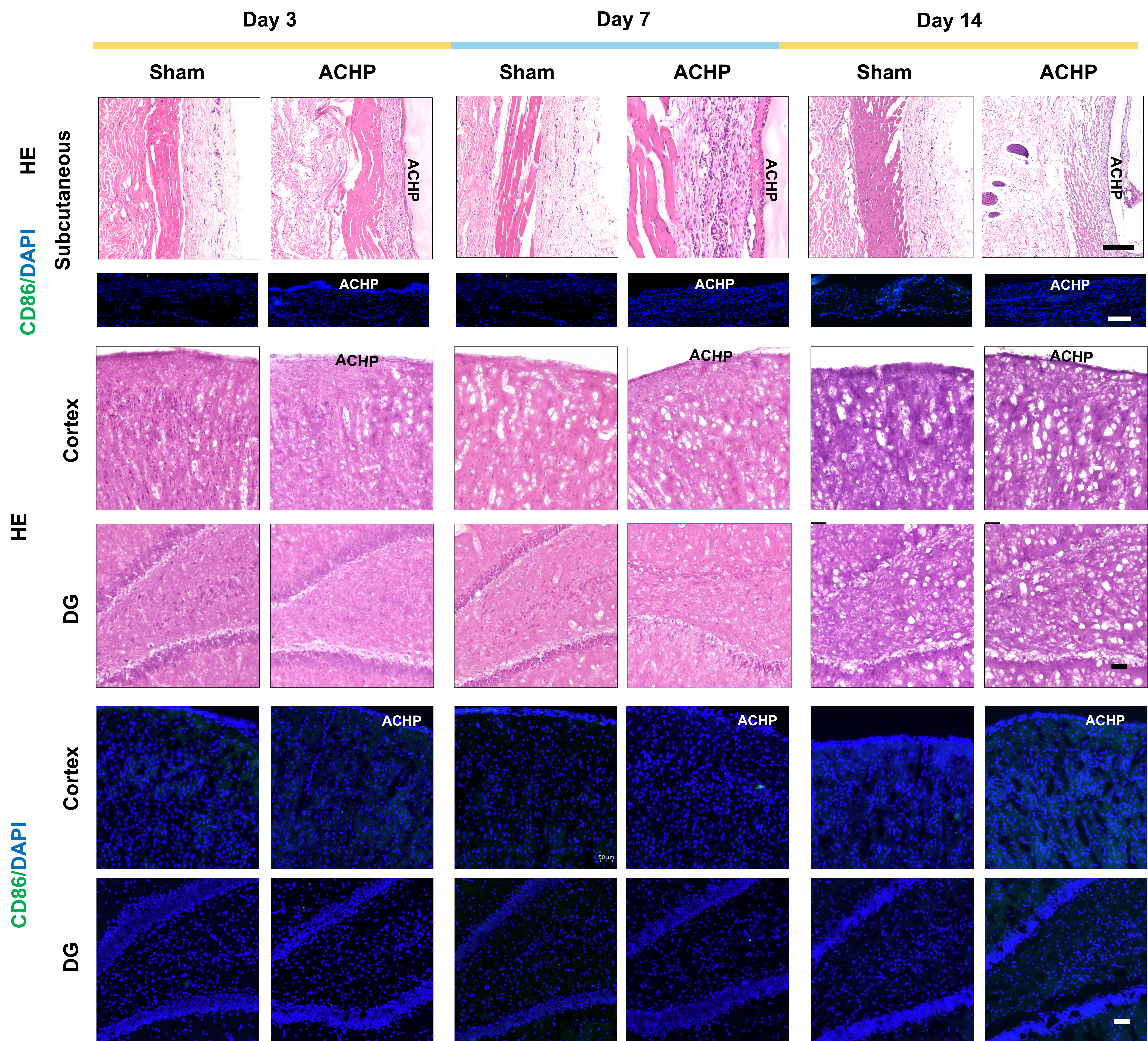


**Figure S17.** Upper panels show the biocompatibility results of ACHP at 3, 7, and 14 days after subcutaneous implantation (HE staining and CD86 staining); lower panels show the biocompatibility results of ACHP at 3, 7, and 14 days after intracerebral implantation (HE staining and CD86 staining).


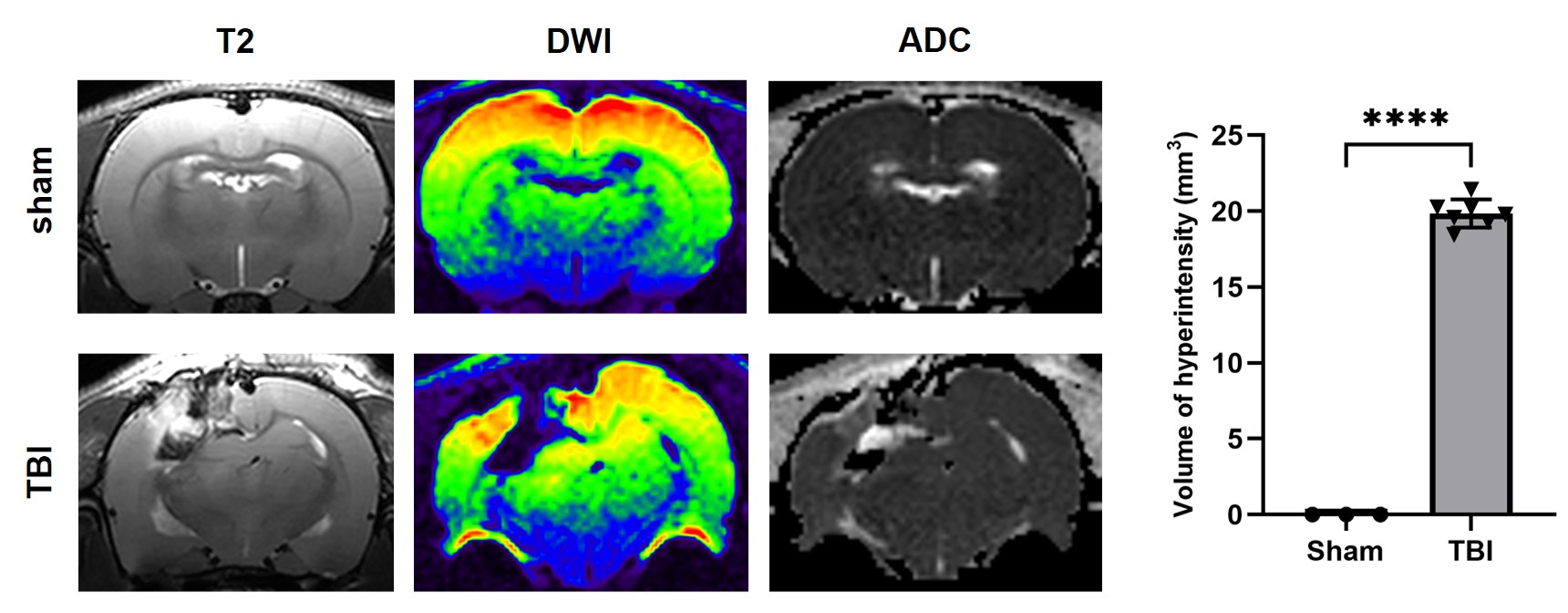


**Figure S18.** Stability of the TBI model: T2-weighted, diffusion-weighted imaging (DWI), and apparent diffusion coefficient (ADC)-weighted magnetic resonance imaging (MRI) images, along with statistics of the lesion area based on T2-weighted imaging. Data are presented as mean ± SD; ****P < 0.0001 vs. Sham group.


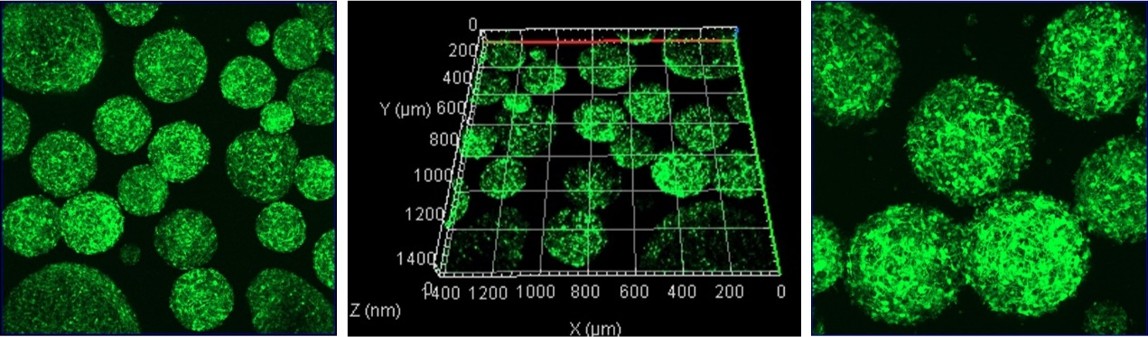


**Figure S19.** GFP labeled NSCs.


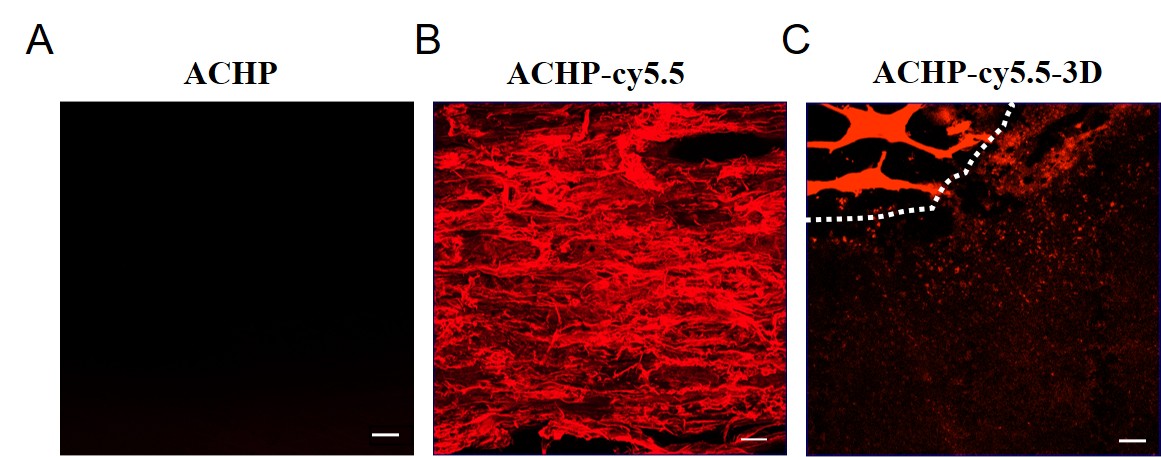


**Figure S20.** **PDA degradation in vivo**. A) No fluorescence signal was detected in the ACHP group, indicating that no fluorescence labeling occurred in this group. B) The ACHP-cy5.5 group exhibited strong red fluorescence, suggesting that the cy5.5 were successfully labeled. C) The ACHP-cy5.5-3D group also showed red fluorescence signals. Within the dashed-line box, the presence of the material can be observed, and it is evident that some PDA has dissociated and infiltrated into the brain tissue. Scale bar, 100 μm.

The amino groups on the surface of ACHP were conjugated to the carboxyl groups on the surface of Cy5.5 through the formation of amide bonds mediated by EDC (1-Ethyl-3-(3-dimethylaminopropyl) carbodiimide) and NHS (N-Hydroxysuccinimide), successfully modifying the surface of ACHP with Cy5.5. Subsequently, ACHP-Cy5.5 was implanted into a rat model of traumatic brain injury (TBI). Two days post-implantation, ultrasound was applied. Tissue samples were collected one day after the ultrasound treatment, cryosection, and the degradation of PDA in vivo was observed.


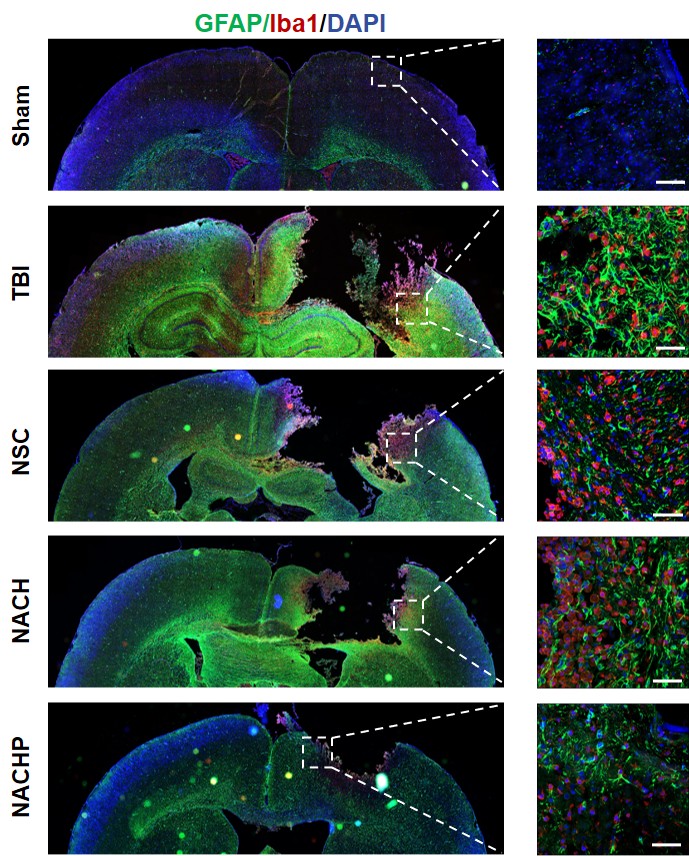


**Figure S21.** Histological Analysis of Inflammatory Response: Immunofluorescence images showing the expression of GFAP (green, astrocyte marker) and Iba1 (red, microglial marker) in coronal brain sections. Treatment groups include Sham, TBI, NSC, NACH, and NACHP. Insets highlight localized areas of inflammation and glial activation. n=6. Scale bar, 50 μm.


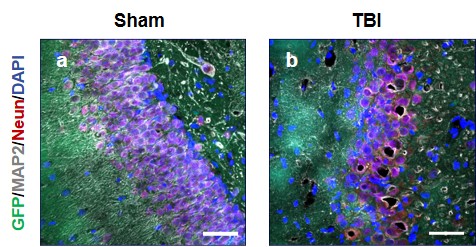


**Figure S22.** Neuronal Regeneration Assessment: Representative images of neuronal markers MAP2 (green) and Neun (red) in the peri-injury region across treatment groups. Enlarged views show detailed neuronal recovery. n=6. scale bar: 50 μm.


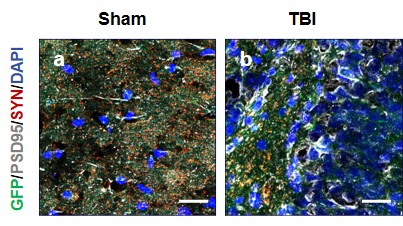


**Figure S23.** The presynaptic marker SYN (red) and the postsynaptic marker PSD95 (white) were stained by immunofluorescence after 28 days. Treatment groups: Sham, TBI. n=6. scale bar: 50 μm


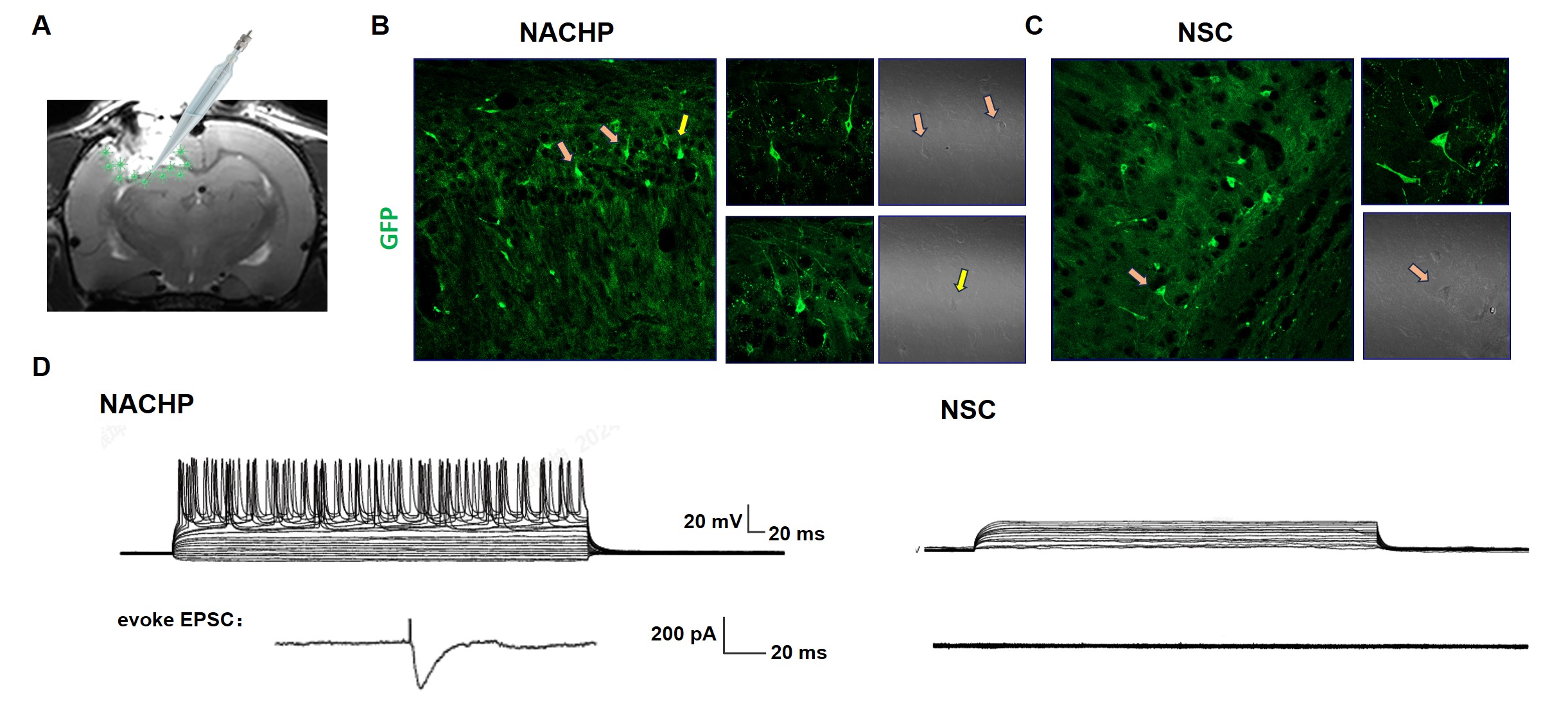


**Figure S24.** Electrophysiological properties of graft-derived cells in the peri-lesion area. (A) Schematic diagram illustrating the experimental setup and recording location. (B) Representative images of GFP-labeled neural stem cells (NSCs) in the NACHP group: GFP fluorescence (left), bright field (middle), and merged view with higher magnification (right). (C) Representative image of GFP-labeled NSCs in the NSC group (control). (D) Patch-clamp recording from a GFP-labeled cell in the NACHP group, showing evoked action potentials (upper panel) and spontaneous excitatory postsynaptic currents (EPSCs; lower panel). Patch-clamp recording from a GFP⁺ cell in the NSC group, showing no detectable action potentials or synaptic currents (right).


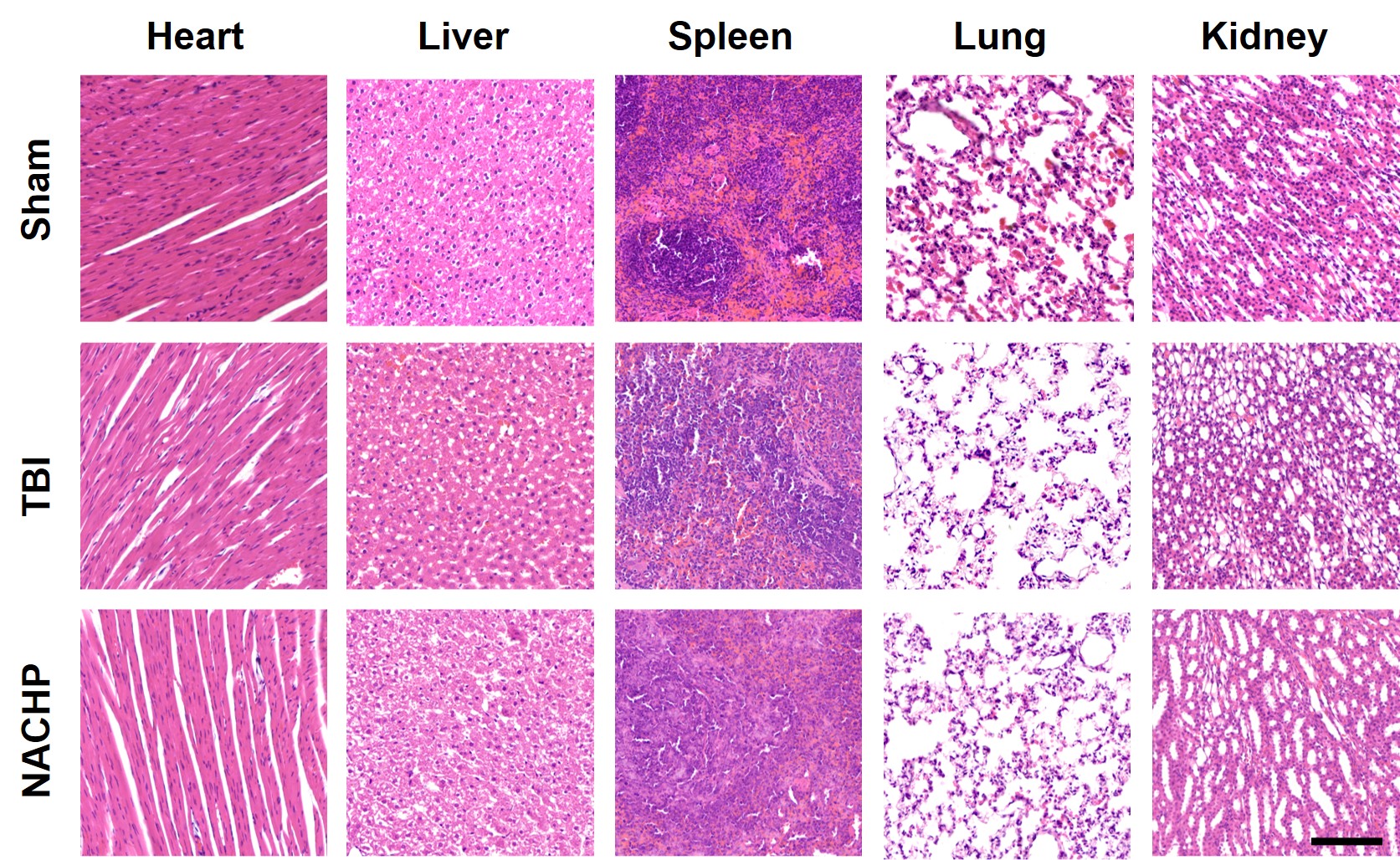


**Figure S25.** Tumorigenic test: HE staining of heart, liver, spleen, lung and kidney after 28 days of TBI treatment. n=6. scale bar: 100 μm


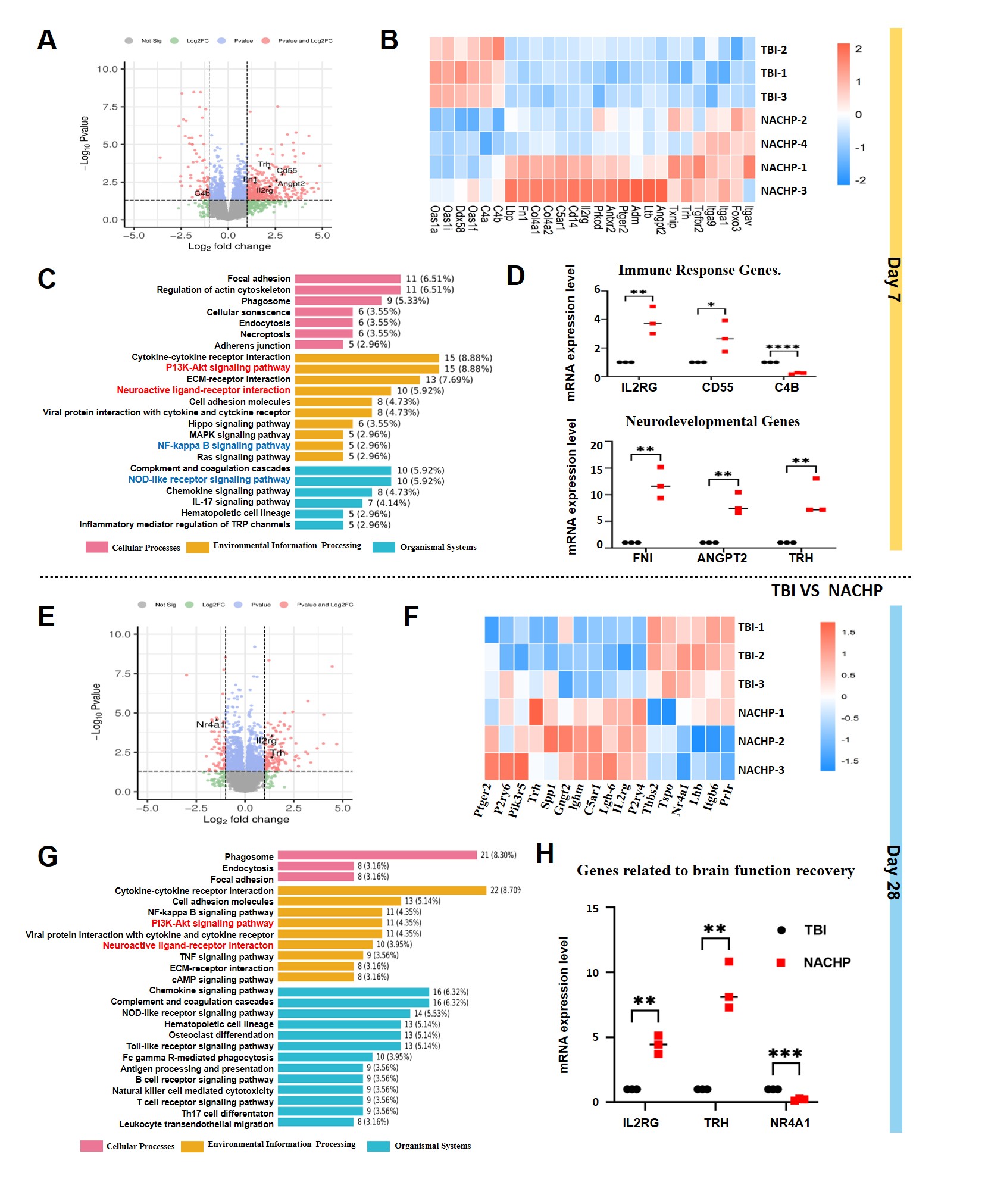


**Figure S26.** Transcriptome sequencing and in vivo validation at 7 and 28 days. (A) Volcano plot of differentially expressed genes (DEGs) between the TBI and NACHP groups at 7 days post-treatment. (B) Heatmap of DEGs related to immune response and neural differentiation/development in the TBI and NACHP groups at 7 days. (C) KEGG enrichment analysis of DEGs at 7 days, categorized into cellular processes, environmental information processing, and organismal systems. (D) qRT-PCR validation of representative genes involved in immune response and neural development at day 7. (E) Volcano plot of DEGs at 28 days post-treatment. (F) Heatmap of genes associated with neural regeneration and functional recovery in the brain in the TBI and NACHP groups at 28 days. (G) KEGG enrichment analysis at 28 days, categorized similarly to (C). (H) qRT-PCR validation of genes related to functional recovery of the brain at 28 days. Statistical significance: *p < 0.05, **p < 0.01, ***p < 0.001.

Table S1. Sequences of RT-qPCR primers

| Gene | Forward primers (5’-3’) | Reverse primers (5’-3’) |
| --- | --- | --- |
| rGAPDH | TGAACGGGAAGCTCACTGG | TCCACCACCCTGTTGCTGTA |
| rTNC | AAAGCAGCCACCCGCTATTAC | GATCTCCTCTGTCAAGACCTCAA |
| rCACNA1B | CCCTGGTGGCATTTGCATTC | AGTTTAGGCAGCCGCTTGAT |
| rGabbr2 | TGGTGCAGCTTTCCTTCGCCG | ACCGCGTTGTCTGACGGCAC |
| rSLC6A11 | gaccgttaatgactgtgagg | ggaaggaaggctggagac |
| rIL2RG | CCGACCAACCTCACTATGCA | GATTCTCTGGAGCCCATGGG |
| rCD55 | TGTGATGCTCTTACTCACTGG | GTATCCATTCTTCCTGGACATTC |
| rC4B | CTTTCTGGTCCGGGCTTCTT | TACTGGGGGTCTCCCTTGAG |
| rFN1 | GGACCAGAGATCTTGGATGTTC | CAAGAGATGGTTGTCTGAGAGAGA |
| rANGPT2 | CCTCGACTACGACGACTCAGT | TCTGCACCACATTCTGTTGGA |
| rTRH | TCTGCAGAGTCTCCACTTCG | AGAGCCAGCAGCAACCAA |
| rNR4A1 | GGGAGATCAAGGCATACCCG | ACCATCAAACCCAGGGACAC |
| mGAPDH | AGGTCGGTGTGAACGGATTTG | GGGGTCGTTGATGGCAACA |
| mIL13 | TGAGCAACATCACACAAGACC | GGCCTTGCGGTTACAGAGG |
| mIL6 | CTGCAAGAGACTTCCATCCAG | AGTGGTATAGACAGGTCTGTTGG |

"r" stands for rat primers, and "m" stands for mouse primers.

**Table S3. The information for primary** **antibodies and secondary antibodies**

| Antibody | Catalog No. | Vendor | Dilution |
| --- | --- | --- | --- |
| Ki67 (Rabbit) | ab16667 | Abcam | 1:500 |
| Nestin (Rabbit) | ab316018 | Abcam | 1:500 |
| PAX6 (Rabbit) | ab195045 | Abcam | 1:500 |
| SOX2 (Rabbit) | ab97959 | Abcam | 1:500 |
| SOX1 (Rabbit) | ab109290 | Abcam | 1:500 |
| MAP2 (Chicken) | ab92434 | Abcam | 1:500 |
| Neun (Rabbit) | ab177487 | Abcam | 1:500 |
| VGLUT1 (Mouse) | 135011 | Synaptic Systems | 1:500 |
| VGAT (Rabbit) | 131002 | Synaptic Systems | 1:200 |
| CD16/32 (Mouse) | 553142 | BD Biosciences | 1:500 |
| CD206 (Rabbit) | 24595S | Cell Signaling Technology | 1:500 |
| PSD95 (Mouse) | ab13552 | Abcam | 1:100 |
| SYN (Rabbit) | ab32127 | Abcam | 1:100 |
| GFAP (Chicken) | ab7260 | Abcam | 1:500 |
| Iba1 (Rabbit) | ab178847 | Abcam | 1:500 |
| Anti-Chicken IgG (Alexa Fluor 488) | ab150169 | Abcam | 1:500 |
| Anti-Chicken IgG (Alexa Fluor 647) | ab150175 | Abcam | 1:500 |
| Anti-Rabbit IgG (Alexa Fluor 488) | ab150077 | Abcam | 1:500 |
| Anti-Rabbit IgG (Alexa Fluor 568) | ab175471 | Abcam | 1:500 |
| Anti-Mouse IgG (Alexa Fluor 488) | ab150113 | Abcam | 1:500 |
| Anti-Mouse IgG (Alexa Fluor 568) | ab175473 | Abcam | 1:500 |
| Anti-Mouse IgG (Alexa Fluor 647) | ab150115 | Abcam | 1:500 |
